# Supplementary material for: Role of feasibility and pilot studies in randomised controlled trials: a cross-sectional study
Source: BMJ Open. 2018 Sep 25;8(9):e022233. doi: 10.1136/bmjopen-2018-022233 (PMC6169762; doi:10.1136/bmjopen-2018-022233)
Supplement: Author's manuscript [file bmjopen-2018-022233.draft_revisions.pdf]

BMJ Open

BMJ Open is committed to open peer review. As part of this commitment we make the peer review history of every article we publish publicly available.

When an article is published we post the peer reviewers' comments and the authors' responses online. We also post the versions of the paper that were used during peer review. These are the versions that the peer review comments apply to.

The versions of the paper that follow are the versions that were submitted during the peer review process. They are not the versions of record or the final published versions. They should not be cited or distributed as the published version of this manuscript.

BMJ Open is an open access journal and the full, final, typeset and author-corrected version of record of the manuscript is available on our site with no access controls, subscription charges or pay-per-view fees (http://bmjopen.bmj.com).

If you have any questions on BMJ Open's open peer review process please email editorial.bmjopen@bmj.com

BMJ Open

The role of feasibility and pilot studies in randomised controlled trials: a retrospective cohort study

Journal:	*BMJ Open*
Manuscript ID	bmjopen-2018-022233
Article Type:	Research
Date Submitted by the Author:	08-Feb-2018
Complete List of Authors:	Blatch-Jones, Amanda; National Institute for Health Research Evaluation, Trials and Studies Coordinating Centre (NETSCC), University of Southampton<br>Pek, Wei; University of Southampton, Faculty of Medicine<br>Kirkpatrick, Emma ; University of Southampton, Southampton Clinical Trials Unit<br>Ashton-Key, Martin; University of Southampton, NETSCC
Keywords:	Randomised Controlled Trials, Pilot studies, Feasibility studies, Health Technology Assessment

SCHOLARONE™
Manuscripts

For peer review only

Title Page

The role of feasibility and pilot studies in randomised controlled trials: a retrospective cohort study

Amanda Blatch-Jones[a*]
Senior Research Fellow

Wei Pek[b]
Medical student, BM5 Medicine

Emma Kirkpatrick[c]
Trial Manager

Martin Ashton-Key[a]
Scientific Director and Consultant in Public Health Medicine

[a]National Institute for Health Research
Evaluation, Trials and Studies Coordinating Centre (NETSCC)
University of Southampton
Alpha House, Enterprise Road
Southampton SO16 7NS
UK

[b] Faculty of Medicine
University of Southampton
Southampton General Hospital
Southampton
UK SO16 6YD

[c] Southampton Clinical Trials Unit
University of Southampton
Southampton General Hospital
Southampton
UK SO16 6YD

*Corresponding author's email address: ajy5@soton.ac.uk

For peer review only

Article summary

Strengths and limitations of this study

- This paper assesses the role of pilot and feasibility studies funded by the NIHR HTA programme
- The study reviews the different ways pilot and feasibility studies funded by the HTA programme and how they inform the design of a trial (review of study elements)
- The study contributes to the limited literature in this area whilst maximising the value and importance to adding value research agenda
- Although the data covers a five year period, the number of eligible studies are small

Acknowledgements: We would like to acknowledge the NIHR HTA Programme for providing data support during the preliminary stage of the study and the NETSCC Reporting Services team for identifying the relevant applications for the cohort. Professor Jane Blazeby for providing external expert advice during the study.

Ethic statement: This study did not require ethics approval as no patient or clinical data were required.

Funding statement: This study was supported by the NIHR Evaluation, Trials and Studies Coordinating Centre through the Research on Research programme as part of a University of Southampton, Faculty of Medicine, BM5 Medicine, 4th Year project. The views and opinions are those of the authors and do not necessarily reflect those of the Department of Health, or of NETSCC. This research received no specific grant from any funding agency in the public, commercial or not-for-profit sectors.

Contribution of Authors: The study was conceived and designed by MAK, ABJ and EK. The work was undertaken by WP as part of a 4th medical student research project, supervised and supported by MAK, ABJ and EK. Analysis and quality assurance was conducted by WP and MAK and the Access database was prepared by ABJ. All authors read and approved the final manuscript. ABJ is guarantor of the study.

Data sharing: Data on the included trials are available on request from the corresponding author.

Licence for publication: The Corresponding Author has the right to grant on behalf of all authors and does grant on behalf of all authors, a worldwide license to the Publishers and its licensees in perpetuity, in all forms, formats and media (whether known now or created in the future), to i) publish, reproduce, distribute, display and store the Contribution, ii) translate the Contribution into other languages, create adaptations, reprints, include within collections and create summaries, extracts and/or, abstracts of the Contribution, iii) create any other derivative work(s) based on the Contribution, iv) to exploit all subsidiary rights in the Contribution, v) the inclusion of electronic links from the Contribution to third party material where-ever it may be located; and, vi) license any third party to do any or all of the above.

Competing interests: The authors have no competing financial interests; however, ABJ and MAK are employed by the University of Southampton to work for NETSCC. ABJ is employed as the Senior Research Fellow for the Research on Research programme and has worked for NETSCC (and its predecessor organisation) in various roles since 2008. MAK is the scientific director at NETSCC and was an editor of the Health Technology Assessment journal. EK is employed by the University of Southampton and works at Southampton's Clinical Trials Unit. EK

For peer review only - http://bmjopen.bmj.com/site/about/guidelines.xhtml

worked for the Research on Research programme during June 2014 to December 2015. WP was a 4th Year BM5 Medicine student at the University of Southampton.

Abstract word count: 298
Main text word count: 3980

For peer review only

For peer review only - http://bmjopen.bmj.com/site/about/guidelines.xhtml

Abstract

Objectives: To assess the value of pilot and feasibility studies to Randomised Controlled Trials funded by the National Institute for Health Research (NIHR) Health Technology Assessment (HTA) programme. To explore the methodological components of pilot and/or feasibility studies and how they go on to inform full RCTs.

Study design: Retrospective cohort study

Setting: Cohort one: Standalone pilot/feasibility studies published in the HTA Journal or accepted for publication. Cohort two: all funded RCT applications funded by the HTA Programme, including reference to an internal and/or external pilot/feasibility study. Both cohorts included studies with fund decision date between 1 January 2010 and 31 December 2014. The methodological components were assessed using an adapted framework from a previous study.

Main outcome measures: The proportion of standalone pilot and feasibility studies which recommended proceeding to full trial and determine what study elements were assessed. The proportion of HTA funded trials which used internal and external pilot and feasibility studies to inform the design of the trial.

Results: Cohort 1 identified 15 standalone pilot/feasibility studies. Study elements most commonly assessed were *testing recruitment* (100% in both groups), *feasibility* (83%, 100%) and *suggestions for further study/investigation* (83%, 100%). Cohort 2 identified 161 HTA funded applications: 59 cited an external pilot/feasibility study where *testing recruitment* (50%, 73%) and *feasibility* (42%, 73%) were the most commonly reported study elements. Ninety-two reported an internal pilot/feasibility study where *testing recruitment* (93%, 100%) and *feasibility* (44%, 92%) were the most common study elements reported.

Conclusions: HTA funded research which is inclusive of pilot and feasibility studies assess a variety of study elements. It is clear that pilot and feasibility studies serve an important role in determining the most appropriate design of a trial. However, caution is required about when it is not appropriate to conduct this type of study.

Keywords: Randomised Controlled Trials, Pilot studies, Feasibility studies, Health Technology Assessment

For peer review only

For peer review only - http://bmjopen.bmj.com/site/about/guidelines.xhtml

INTRODUCTION

Pilot and feasibility studies have an important role to play in the development of Randomised Controlled Trials (RCTs). If appropriately used, pilot and feasibility studies can provide sufficient methodological evidence about the design planning and justification of a trial. They are often undertaken to inform elements of the main trial design, but they can also be used to reduce or eliminate problems that limit the successful delivery of trials. In 2009, the Lancet published a paper that highlighted the extent to which research is wasted, and that loss is as much as 85% of research investment.[1] Given the cost and time of investment from researchers and major health research funders, there is now a growing demand to assess and examine the factors associated with how research is wasted.[2] Poor research design has been associated with factors such as non-reference to a pre-existing systematic literature review or bias generated by inadequate concealment of treatment allocation.[1] Much attention has focused on the design, conduct and analysis of clinical research, primarily since much of the wastage is as a result of poor and inadequate methods.[3]

Over the last ten years, we have seen how pilot and feasibility studies have become an important feature in terms of gathering evidence to inform the development of a full trial. Conducting a pilot or feasibility study to determine any uncertainties prior to the main trial may help to eradicate issues and thus inform the definitive trial. More importantly perhaps, is the role pilot and feasibility studies can have in modifying the design and conduct, and therefore increasing the value of the research, helping to avoid methodological design flaws and reducing the burden of research waste.

Despite the growing importance of pilot and feasibility studies there is still a lack of clarity about the use of the two terms.[4-6] In 2008, the Medical Research Council (MRC) published guidance on developing and evaluating complex interventions to demonstrate the value and importance of pilot and feasibility studies as a key element in the development and evaluation process. However, the guidance did not attempt to explain or provide any definition for the terms 'pilot' and 'feasibility'.[6] It was not until two years later that Thabane *et al.* reviewed the key aspects of pilot studies and provided a detailed account of pilot studies which included a number of definitions.[5] Around the same time (2009), the National Institute for Health Research, Evaluation, Trials and Studies Coordinating Centre (NETSCC) published a support document detailing what feasibility and pilot studies are.

- Feasibility studies are defined as "pieces of research done before a main study in order to answer the question "Can this study be done?". They are used to estimate important parameters that are needed to design the main study…feasibility studies do not evaluate the outcome of interest."
- Pilot studies are defined as "a version of the main study that is run in miniature to test whether the components of the main study can all work together. It is focused on the processes of the main study…it will therefore resemble the main study in many respects."

(https://www.nihr.ac.uk/funding-and-support/documents/funding-for-research-studies/research-programmes/PGfAR/Feasibility%20and%20Pilot%20studies.pdf – accessed 06/02/2018).

These definitions have gone some way to aid the understanding for when it is appropriate to do pilot or feasibility studies as part of the definitive trial. These definitions are now widely used across NIHR.

Despite the importance of the role of pilot and feasibility studies in informing RCTs, there is little empirical evidence about the use of these studies in informing future trials. For example, the Lancet series in 2014 did not make reference to the usefulness of pilot and feasibility studies in the context of increasing value and reducing waste in research design, conduct or analysis.[3]

For peer review only - http://bmjopen.bmj.com/site/about/guidelines.xhtml

Lancaster *et al.* and Arain *et al.* provided a methodological framework to assess how pilot studies are used to inform the conduct and reporting of pilot studies.[4 7] Both described the challenges and complexities in the reporting of pilot studies. Arain *et al.* further explored these complexities in relation to feasibility studies and full trials.

The aim of this study is to contribute evidence to this important gap in the current literature. The purpose of this paper is to describe the process and results of how, and in what way, pilot and feasibility studies have been used to inform full RCTs.

For peer review only

For peer review only - http://bmjopen.bmj.com/site/about/guidelines.xhtml

METHODS

The Health Technology Assessment (HTA) Programme has a long history of commissioning pilot and feasibility studies. Therefore, the published reports of standalone pilot and feasibility studies were examined to determine which elements of research design are most often assessed. Applications for funded HTA trials were also assessed to establish how full trials are informed by previously completed pilot / feasibility studies as well as pilot studies embedded within the trial.

Data source

An assessment of the NIHR HTA Programme over a five-year period (2010-2014) was conducted using two retrospective cohorts. In order to identify the included studies for both cohorts we

1. Firstly reviewed the project / journal title. If it was not possible to confirm the inclusion of a pilot / feasibility study;
2. We then assessed the abstract, to confirm the citation of or main study type as pilot / feasibility study. If it was still not possible to determine;
3. We then reviewed the full Journal article or application form

Cohort 1: Standalone pilot and feasibility studies

Standalone pilot / feasibility studies funded by the HTA Programme with a fund decision date from 01 January 2010 to 31 December 2014, which have published in the HTA Journal or are currently being prepared for publication and have been signed off by the editors (only those in production were included). The published Journal/approved final version of the published report was used as the source for data extraction. The standalone studies were categorised into 'pilot study', 'feasibility study' or 'both'.

Cohort 2: Randomised Controlled Trials

Trials funded through the HTA Programme with a fund decision date between 01 January 2010 to 31 December 2014. The application form of a funded trial was used as the source for data extraction. The trials were categorised based on the type of pilot and/or feasibility: 'external / previous pilot study', 'external / previous feasibility study', 'internal pilot study', 'internal feasibility study' or 'other (mixed study)'.

NETSCC's research management databases were used to identify the two cohorts. Search terms were used to search for relevant data to ensure the feasibility of future replications. The key search terms used were: pilot, feasibility, preliminary work, earlier/previous study.

In addition to the search using key terms, a targeted search was carried out on specific areas of the application form. Focusing on specific areas of the application was relevant in identifying where the elements of the study design would most likely be described in relation to the pilot and/or feasibility study.

Classification systems

The definition of pilot and feasibility studies agreed by NIHR for four Programmes was used for the purpose of this study (see: https://www.nihr.ac.uk/funding-and-support/documents/funding-for-research-studies/research-programmes/PGfAR/Feasibility%20and%20Pilot%20studies.pdf). These definitions were also used by Arain *et al.*[7]

The classification systems developed by Arain *et al.* and Bugge *et al.* were adapted to determine what elements of a study design were assessed or used to inform the full trial (see *Table 1*).[7 8] In both cohorts, the elements of the study design were examined in terms of

For peer review only - http://bmjopen.bmj.com/site/about/guidelines.xhtml

a. Did the study explicitly state it assessed any of these elements? (yes/no)
b. Were there any recommended changes as a result of the assessment? A yes response was defined as: the authors reported a change / recommendation to be considered but did not necessarily report what that change was. If the authors did not explicitly state a recommendation, it was assumed that no changes were required.

The text pertaining to the pilot and/or feasibility study was also extracted for quality assurance purposes.

Insert Table 1 here

Two additional study elements were included in cohort two which were not reported in cohort one. These were '*delivery of intervention*' and '*testing/developing materials*'.

Piloting

Data extraction tables for cohort 1 and cohort 2 were piloted with an initial sample of 10 studies. No changes were required to the classification system previously adopted by Arain *et al.* as a result of the pilot work.

Data quality and assurance

Our approach to quality assurance was guided by the Strengthening the Reporting of Observational Studies in Epidemiology (STROBE), which although designed for observational studies, could be applied to the processes we used in this current study.

For both cohorts, WP extracted all data and a second person assessed and reviewed the data to ensure the accuracy of data extraction. All of cohort 1 was assessed followed by 15% of cohort 2 (purposive sampling of 5% of the cohort followed by 10% randomly selected application forms). The remaining 85% was subsequently reviewed by MAK to determine the reliability and validity of the data extraction and usability of the adapted template. All disagreements were discussed by the team and were resolved by consensus. Data management was undertaken by WP with support from ABJ.

Data analysis

The classification system adapted from Arain *et al.* was captured using Microsoft Access.[7] A separate Access form was developed to extract data independently for both cohorts. Both cohorts were exported into Microsoft Excel and then subsequently into IBM SPSS V.22. Excel was used to calculate the median and the inter-quartile range for cohort 1 only. Data were analysed and interpreted using descriptive and inferential statistics to determine the frequency of the study design elements and how often changes were recommended for full trials.

For peer review only - http://bmjopen.bmj.com/site/about/guidelines.xhtml

Results

In cohort 1 we identified 47 published standalone pilot and/or feasibility studies and in cohort 2 we identified 303 HTA funded RCTs during the five-year period (01 January 2010 – 31 December 2014). Fifteen standalone studies were identified and eligible for cohort 1 and 161 funded HTA applications were identified and eligible for cohort 2.

Cohort 1

A total of 47 standalone studies were identified. Thirty-two were excluded on further examination due to not being a pilot or feasibility study. The remaining 15 studies were categorised into three separate groups (see *Figure 1*). We found that 13 of the 15 study elements included in the adapted framework were assessed in standalone pilot studies compared to nine study elements in feasibility studies.

In this cohort, it was found that seven studies used the terms "pilot" and "feasibility" interchangeably and it was difficult to determine, even with the NIHR definition, what type of study was undertaken. Therefore, it was not possible to accurately determine which study elements belonged to which, and in some cases the authors described the conduct of both pilot and feasibility work. The team agreed to combine pilot and feasibility together in this instance, which was also found in Arain *et al*.

Insert Figure 1 here

The median number of participants for the standalone studies (n=15) was 46, with an inter-quartile range 29 to 98. Of the 15 eligible standalone pilot and/or feasibility studies, the most commonly reported study design element was *testing recruitment*. In all three groups (pilot studies, feasibility studies and pilot/feasibility studies), all 15 studies assessed recruitment (6/6, 2/2 and 7/7 respectively) (see *Table 2*). Half of these also reported recommended changes to recruitment (3/6, 1/2 and 3/7 respectively). Interestingly, both feasibility studies only (2/2) and pilot/feasibility groups (7/7) assessed the need for *further study* and suggested recommended changes (*further study* referred to whether further investigation was required using a large RCT and where future trial data could be of benefit).

Insert Table 2 here

Cohort 2

A total of 303 HTA funded applications were identified. Eighty-two were excluded upon examination as they were not RCTs (for example cohort studies, diagnostic accuracy test studies) and a further 60 applications were excluded due to not being informed by any external or internal pilot and/or feasibility study. The remaining 161 applications were reviewed and subsequently grouped into five categories (see *Figure 2*). As the application was used as the source of data extraction, the outcome of the internal pilot/feasibility study was not available (n=92). Not all citations included the number of participants in the external pilot / feasibility study and we did not go back to the originally cited reference (n=59). Therefore, it was not appropriate to estimate the median or inter quartile range for this cohort.

The *others* group comprised of applications that were informed by a combination of more than one preliminary study (e.g. internal and/or external pilot study and/or feasibility study). Of those 10 applications,

- Six of the ten were informed by external pilot studies,

For peer review only - http://bmjopen.bmj.com/site/about/guidelines.xhtml

- Seven of the ten were informed by external feasibility studies,
- Seven of the ten were informed by internal pilot studies and
- One of the ten was informed by an internal feasibility study.

No further analysis was conducted on these 10 applications due to the diverse nature of the study types in this subgroup.

Insert Figure 2 here

External pilot and feasibility studies

Of the 161 applications, 29.8% (48/161) reported or cited a previous external pilot study not recently done by the applicant and 6.8% (11/161) reported an external feasibility study. For this subset, all of the study elements (n=17) were assessed by external pilot studies but no single study assessed all 17 elements. By comparison, 13 of the 17 study elements were assessed by external feasibility studies (see *Table 3*).

In terms of the study elements, *testing recruitment*, *determining the sample size and numbers available*, and the *feasibility* were the most commonly reported criteria assessed in both external pilot and feasibility studies (see *Figure 3*). The number of reported recommended changes based on the results of the external pilot or feasibility study were however minimal. Although, in some applications it was possible to detect a change, the authors did not explicitly state a recommended change. Therefore, it was not possible to determine whether this was based on the pilot or feasibility study, or some other factor.

Insert Table 3 here

Insert Figure 3 here

Internal pilot and feasibility studies

Of the 161 applications, 49.7% (80/161) reported an internal pilot study and 7.5% (12/161) reported an internal feasibility study. Due to the source of data extraction (the application form) it was not possible to determine whether the funded internal pilot or feasibility study had made any recommended changes: as the internal study had not yet been conducted.

For the internal studies, we found 14 of the 17 study elements were being assessed by internal pilot studies compared to 10 study elements in feasibility studies. Based on assessment only, the most common study element to be reported was *testing recruitment* (74/80 and 12/12 respectively) and *feasibility* (35/80 and 11/12 respectively) for both internal pilot and feasibility study (see *Table 4*). As *Figure 4* shows, there were several similarities between a number of study elements assessed by both pilot and feasibility studies.

Insert Table 4 here

Insert Figure 4 here

For peer review only - http://bmjopen.bmj.com/site/about/guidelines.xhtml

Discussion

This study found that pilot and feasibility studies do play a role in the development and design of definitive RCTs. In both cohorts it was clear that two study elements were commonly assessed: t*esting recruitment* and *feasibility*. This has important implications for the success of a trial, given that many trials struggle with recruitment and often request extensions or become at risk of closure.[9 10] Our findings showed how trials use pilot and/or feasibility studies in an attempt to assess and evaluate prior to a full trial, whether it is likely to be able to recruit its target sample size and whether the study is indeed feasible as a full trial. In both cohorts, we found pilot studies assessed more study elements than feasibility studies. This also applied to the internal and external study groups in cohort 2; external and internal pilot studies were used to assess more study elements than that of feasibility studies.

Strengths and weaknesses of the study and in relation to other studies

The main strength of the study was the inclusion of all HTA funded studies over a five-year period. Although cohort one (standalone) only included 15 studies, this was as expected. For this cohort, we identified an increase in almost all of the study elements being assessed compared to earlier work by Arain *et al.*[7] In cohort two, over half of the HTA funded applications included a pilot and/or feasibility study (internal and/or external) (161/303). Compared to Arain *et al.*[7] the findings were similar for the external pilot and feasibility studies cited in terms of the number of study elements assessed and the number of studies included. For example, *testing recruitment* was the most frequently reported element for pilot studies in both the current study and Arain *et al.*, and d*etermining the sample size and the numbers available* was identical in both studies. However, *randomisation*, *clinical outcomes* and *feasibility* were reported more frequently by Arain *et al.* than the current study. For the external feasibility studies, the current study found more study elements being assessed than that of Arian *et al.* in terms of *testing recruitment*, *determining the sample size and the numbers available*, *randomisation*, *acceptability*, *feasibility* and *follow up/drop out*.

For the internal pilot studies, similar findings were found when comparing Arain *et al.* to the current study: d*etermining the sample size and the numbers available, randomisation* and *clinical outcomes* were assessed more in Arain *et al.* than the current study. As with the internal feasibility studies, we found the current study to report more study elements being assessed than that of Arain *et al.*: *testing recruitment*, *determining the sample size and the numbers available*, *follow up/drop out*, *randomisation*, *acceptability* and *feasibility*. These differences, particularly found with the feasibility studies could be associated with changes over time in the use and understanding of feasibility studies.

This study relied on an adapted version of the Arain *et al.* framework. As some of the study elements were expanded and new ones were added a direct comparison with Arain *et al.* findings is limited.[7] Given the subjective nature of some of the study elements, we chose to quality assure all data to eradicate and reduce any known errors. Since the analysis was based explicitly on the reporting of the applicants, and did not include any subjective account or interpretation of what was reported, we may have under reported the number of study elements assessed and/or recommended.

We also noted a mismatch in numbers between those assessing study elements and those where recommendations were made in cohort one. This was due, in part, to how each study element was reported by the applicants. For example if a study did not specify that they had assessed these elements but made recommendation for changes, we only inferred that they assessed it, but it could not be recorded in the data, hence the mismatch in the findings. This

For peer review only - http://bmjopen.bmj.com/site/about/guidelines.xhtml

does however highlight the importance of clearly reporting how, what and where the pilot and/or feasibility study had an impact on the design of the definitive trial.

Implications

The level of appropriateness in the reporting of pilot and feasibility studies could largely be affected by the lack of clarity and awareness of the different study requirements. Despite the growing literature on improving the quality of research to reduce waste in research, there is limited literature pertaining to how pilot and feasibility studies fit into this agenda for change. From what literature there is on pilot and feasibility studies, there is still some confusion about when, why and how it is appropriate to conduct a pilot and/or feasibility study. The findings in this study, even with the use of a well-defined definition by NIHR, we still found evidence where applicants did not adhere to the HTA definitions for "pilot" and "feasibility" study on research applications. The terminology is still being used interchangeably. Although the commentary on pilot studies by Thabane *et al.* gives a detailed account of the appropriateness of why and how to conduct a pilot study, a comparison with feasibility studies is lacking.[5] It would be helpful to have a more formal distinction between these two terminologies as suggested by Arain *et al.* A recent study by Eldridge *et al.* goes some way to rectify this by developing a conceptual framework for defining pilot and feasibility studies.[11] The conceptual framework shows promising results, by being compatible with the MRC guidance on complex interventions,[6] and their descriptor of pilot studies is similar to that of the NIHR (NIHR definition). However, it is important to note that the Eldridge *et al.* conceptual framework is slightly different to that adopted by the NIHR.[11]

Having clear definitions of when to use pilot and feasibility studies is clearly important for confirming the progression to a full trial. However, it is also imperative to note the limitations of the use of pilot and feasibility studies and when it is not appropriate to conduct this type of study. Pilot and feasibility studies are to assist and direct the design stage of a trial, they should not be used to assess effectiveness, make claims about whether the treatment works or not, or to perform sample size calculations. They should be explicitly defined as a pilot or feasibility study in the study protocol. Both pilot and feasibility studies have a role to play and are extremely important to the design stage of a trial. However, how they are reported and in what context, requires caution especially when interpreting the findings and delivering a definitive trial.[12]

Conclusion and recommendations

HTA funded research which is inclusive of pilot and feasibility studies are very likely to assess a variety of study elements, which have been evidence-based through this current study using an adapted version of Arain *et al.* framework.[7] However, not reviewing the impact of the preliminary work once the trial commences, we have no way of knowing whether the pilot and/or feasibility studies recommendations were instrumental in the successful completion of the trial. If we are able to demonstrate the value of pilot and feasibility studies we need to place greater emphasis on not only their role in the design stage of a trial but also how this preliminary work predicted favourably, or not, to the completion of the definitive trial. The internal pilot and/or feasibility studies reported in cohort 2 could be used for the basis of continual work in this area. By following up on this cohort we would be able to analyse the successful delivery of the definitive trial and whether the preliminary work had any bearing on this success.

For peer review only - http://bmjopen.bmj.com/site/about/guidelines.xhtml

Recommendations include a larger sample of studies across other UK health research funding agencies to determine the frequency and importance of those study elements reported here. A further assessment between the study elements noted in the pilot and feasibility studies and how this impacted on the eventual design and conduct of the definitive trial would certainly add value. This could be achieved by prospectively evaluating the ongoing use of pilot and feasibility studies in cohort two (specifically the internal pilot and/or feasibility studies) as well as future funded applications to the HTA programme. Highlighting the need for better reporting of pilot and feasibility studies should be regarded as relevant to all research funding bodies. And as such, better guidelines for the design, conduct, analysis and reporting of pilot and feasibility studies are still needed.

Future work could therefore include widening the study outcomes presented here to other NIHR funded programmes such as Programme Grants for Applied Research (PGfAR). Funders might want to consider the use of Arain *et al.* framework when considering the funding of pilot / feasibility studies. Thus, maximising the benefit of research and reducing the extent to which research is wasted. If we find ways to appropriately address the flaws detected at the design and conduct stages of research, then we could start to see how research adds value and reduces the amount of research waste. In order to achieve this, we need clearly defined terminology which is inclusive of funding agencies and researchers' perspective; empirical evidence on the reporting and appropriate use of pilot and feasibility studies, in terms of favourable study elements and; evaluation of the contribution to definitive trial outcomes.

For peer review only

For peer review only - http://bmjopen.bmj.com/site/about/guidelines.xhtml

Table 1: Elements of a study design adapted from Arain *et al.*

**Methods related**
Testing Recruitment
Determining the sample size / numbers available
Follow up / dropout
Hypothesis testing
Resources
Randomisation
Blinding
Outcome measures
Control group
Data collection
Further study suggested
**Intervention related**
Dose / efficacy / safety
Clinical outcomes
Acceptability
Feasibility
In addition to the above, Cohort 2 included:
Delivery of the intervention
Testing/developing materials

For peer review only

For peer review only - http://bmjopen.bmj.com/site/about/guidelines.xhtml

Table 2: Cohort1 - Study elements captured in pilot studies, feasibility studies and pilot/feasibility studies

	**Pilot studies only (n=6)**			**Feasibility studies only (n=2)**			**Pilot/Feasibility studies (n=7)**		
**Criteria**	**Assessed (A): Number (%)**	**Recommended changes (RC): Number (%)**	**A and RC: Number (%)**	**Assessed (A): Number (%)**	**Recommended changes (RC): Number (%)**	**A and RC: Number (%)**	**Assessed (A): Number (%)**	**Recommended changes (RC): Number (%)**	**A and RC: Number (%)**
Testing recruitment	6 (100.0)	3 (50.0)	3 (50.0)	2 (100.0)	1 (50.0)	1 (50.0)	7 (100.0)	3 (42.9)	3 (42.9)
Determining SS and n available	5 (83.3)	1 (16.6)	0	1 (50.0)	2 (100.0)	1 (50.0)	5 (71.4)	1 (14.3)	1 (14.3)
Follow up/dropout	4 (66.6)	3 (50.0)	3 (50.0)	0	1 (50.0)	0	5 (71.4)	2 (28.6)	1 (14.3)
Hypothesis testing	2 (33.3)	0	0	1 (50.0)	0	0	1 (14.3)	0	0
Resources	4 (66.6)	3 (50.0)	2 (33.3)	2 (100.0)	0	0	5 (71.4)	1 (14.3)	1 (14.3)
Randomisation	4 (66.6)	0	0	0	0	0	6 (85.7)	1 (14.3)	1(14.3)
Blinding	0	0	0	0	0	0	2 (28.6)	0	0
Outcome measures	5 (83.3)	4 (66.6)	4 (66.6)	2 (100.0)	1 (50.0)	1 (50.0)	6 (85.7)	4 (57.1)	3 (42.9)
Control group	0	0	0	0	0	0	1 (14.3)	0	0
Data collection	3 (50.0)	0	0	0	1 (50.0)	0	3 (42.9)	1 (14.3)	0
Clinical outcomes	3 (50.0)	2 (33.3)	1 (16.6)	1 (50.0)	0	0	3 (42.9)	0	0
Dose/efficacy/ safety	2 (33.3)	1 (16.6)	1 (16.6)	0	0	0	2 (28.6)	0	0
Acceptability	4 (66.6)	0	0	1 (50.0)	0	0	6 (85.7)	0	0
Feasibility	5 (83.3)	0	0	2 (100.0)	0	0	7 (100.0)	0	0
Suggests further study	5 (83.3)	4 (66.6)	4 (66.6)	2 (100.0)	2 (100.0)	2 (100.0)	7 (100.0)	7 (100.0)	7 (100.0)
Median number of participants (IQR)	47.5 (39.25-85, being equal to 45.75)	NA	NA	14 (7-21, being equal to 14)	NA	NA	58 (35.5-173, being equal to 137.5)	NA	NA

For peer review only - http://bmjopen.bmj.com/site/about/guidelines.xhtml

Table 3: Cohort 2 - Study elements captured in external pilot and feasibility studies

Criteria	External pilot study (n=48)		External feasibility study (n=11)	
	Assessed Number (%)	Recommended changes Number (%)	Assessed Number (%)	Recommended changes (n)
Testing recruitment	24 (50.0)	3 (6.3)	8 (72.7)	0
Determining SS and n available	24 (50.0)	1 (2.1)	4 (36.4)	1 (9.1)
Follow up/dropout	16 (33.3)	0	3 (27.3)	0
Hypothesis testing	10 (20.8)	0	2 (18.2)	0
Resources	2 (4.2)	0	1 (9)	0
Randomisation	7 (14.6)	0	3 (27.3)	0
Blinding	4 (8.3)	1 (2.1)	0	0
Outcome measures	10 (20.8)	1 (2.1)	1 (9.1)	0
Control group	3 (6.3)	0	0	0
Data collection	6 (12.5)	0	2 (18.2)	0
Clinical outcomes	12 (25.0)	0	1 (9.1)	0
Dose/efficacy/safety	14(29.2)	1 (2.1)	0	0
Acceptability	17 (35.4)	0	4 (36.4)	0
Feasibility	20 (41.7)	0	8 (72.7)	0
Suggests further study	8 (16.6)	1 (2.1)	1 (9.1)	0
Delivery of intervention	8 (16.6)	2 (4.2)	0	0
Testing/developing materials	3 (6.3)	0	1 (9.1)	0

Table 4: Cohort 2 - Study elements captured in internal pilot and feasibility studies

Criteria	Internal pilot study (n=80) Assessed No. (%)	Internal feasibility study (n=12) Assessed No. (%)
Testing recruitment	74 (92.5)	12 (100.0)
Determining SS and n available	21 (26.3)	4 (33.3)
Follow up/dropout	28 (35.0)	5 (41.7)
Hypothesis testing	0	0
Resources	3 (3.8)	1 (8.3)
Randomisation	27 (33.8)	4 (33.3)
Blinding	2 (2.5)	0
Outcome measures	16 (20.0)	2 (16.7)
Control group	0	0
Data collection	21 (26.3)	2 (16.7)
Clinical outcomes	1 (1.3)	0
Dose/efficacy/safety	5 (6.3)	1 (8.3)
Acceptability	21 (26.3)	7 (58.3)
Feasibility	35 (43.8)	11 (91.7)
Suggests further study	0	0
Delivery of intervention	7 (8.8)	0
Testing/developing materials	7 (8.8)	0

For peer review only - http://bmjopen.bmj.com/site/about/guidelines.xhtml

References

1. Chalmers I, Glasziou P. Avoidable waste in the production and reporting of research evidence. *The Lancet* 2009;374(9683):86-89. doi: doi: DOI: 10.1016/S0140-6736(09)60329-9
2. Moher D, Glasziou P, Chalmers I, et al. Increasing value and reducing waste in biomedical research: who's listening? *The Lancet* 2016;387(10027):1573-86. doi: https://doi.org/10.1016/S0140-6736(15)00307-4
3. Ioannidis JP, Greenland S, Hlatky MA, et al. Increasing value and reducing waste in research design, conduct, and analysis. *Lancet* 2014;383(9912):166-75. doi: 10.1016/S0140-6736(13)62227-8
4. Lancaster GA, Dodd S, Williamson PR. Design and analysis of pilot studies: recommendations for good practice. *Journal of Evaluation in Clinical Practice* 2004;10(2):307-12. doi: 10.1111/j..2002.384.doc.x
5. Thabane L, Ma J, Chu R, et al. A tutorial on pilot studies: the what, why and how. *BMC medical research methodology* 2010;10(1):1.
6. Craig P, Dieppe P, Macintyre S, et al. Developing and evaluating complex interventions: The new Medical Research Council guidance. *International Journal of Nursing Studies* 2013;50(5):587-92. doi: 10.1016/j.ijnurstu.2012.09.010
7. Arain M, Campbell MJ, Cooper CL, et al. What is a pilot or feasibility study? A review of current practice and editorial policy. *BMC medical research methodology* 2010;10:67. doi: 10.1186/1471-2288-10-67
8. Bugge C, Williams B, Hagen S, et al. A process for Decision-making after Pilot and feasibility Trials (ADePT): development following a feasibility study of a complex intervention for pelvic organ prolapse. *Trials* 2013;14:353. doi: 10.1186/1745-6215-14-353
9. Raftery J, Young A, Stanton L, et al. Clinical trial metadata: defining and extracting metadata on the design, conduct, results and costs of 125 randomised clinical trials funded by the National Institute for Health Research Health Technology Assessment programme. *Health Technology Assessment* 2015;19(11):1-166.
10. Bower P, Brueton V, Gamble C, et al. Interventions to improve recruitment and retention in clinical trials: a survey and workshop to assess current practice and future priorities. *Trials* 2014;15(1):399.
11. Eldridge SM, Lancaster GA, Campbell MJ, et al. Defining feasibility and pilot studies in preparation for randomised controlled trials: development of a conceptual framework. *PLoS One* 2016;11(3):e0150205.
12. Vogel S, Draper-Rodi J. The importance of pilot studies, how to write them and what they mean. *International Journal of Osteopathic Medicine* 2017;23:2-3. doi: 10.1016/j.ijosm.2017.02.001

For peer review only - http://bmjopen.bmj.com/site/about/guidelines.xhtml

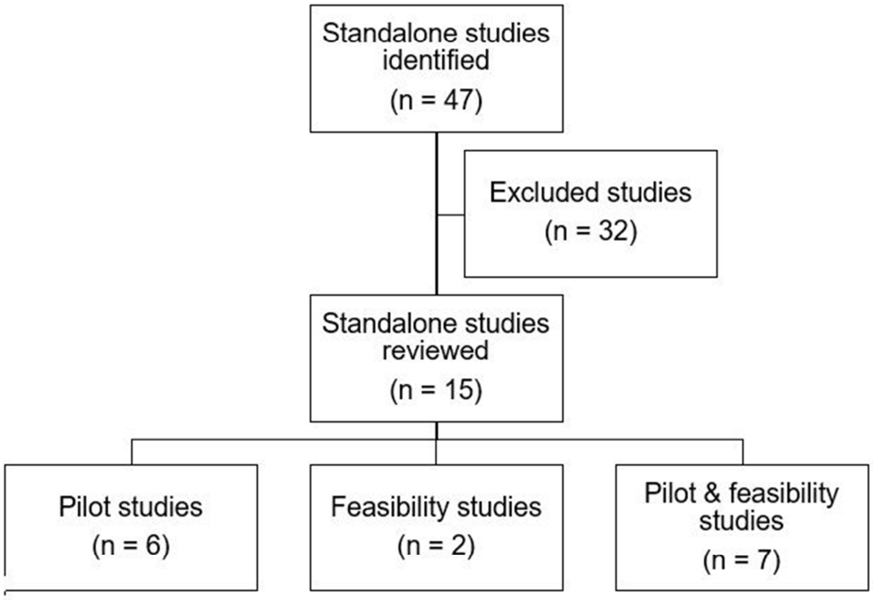

Figure 1: The number of studies identified, excluded and categorised for Cohort 1

Figure 1

129x93mm (144 x 144 DPI)

For peer review only - http://bmjopen.bmj.com/site/about/guidelines.xhtml

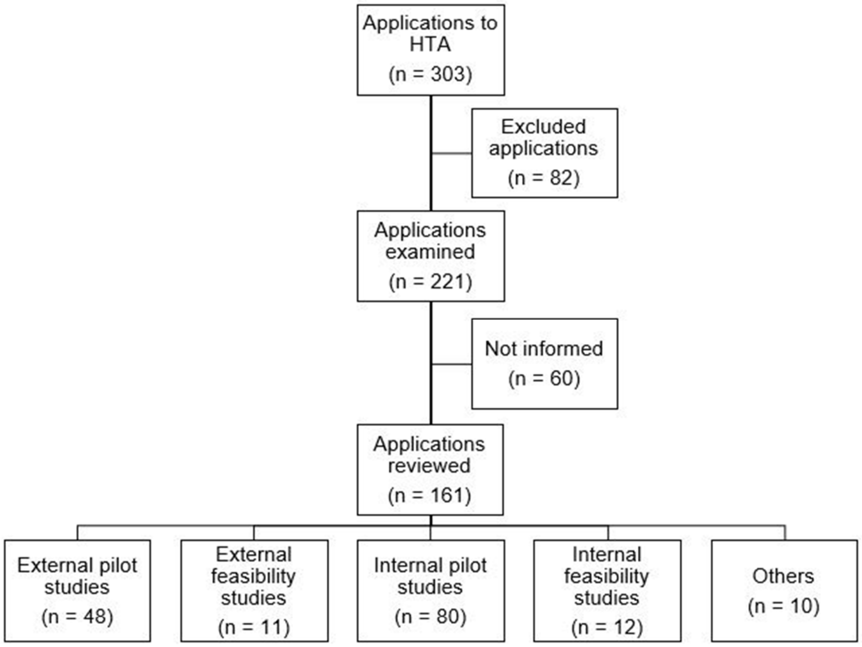

Figure 2: Flow chart showing the number of HTA funded applications for Cohort 2

Figure 2

131x103mm (144 x 144 DPI)

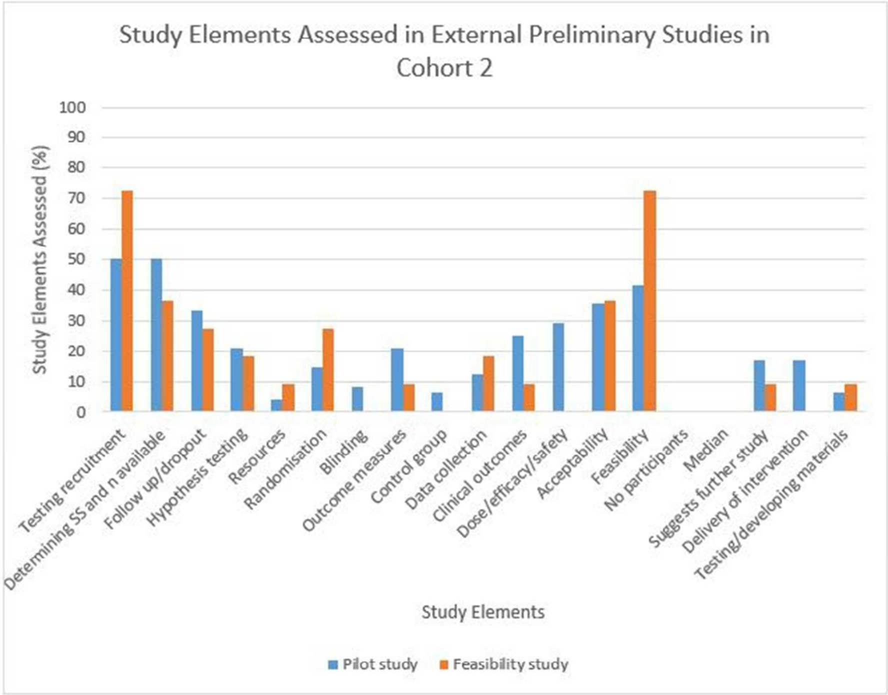

Figure 3: Study elements assessed in external preliminary studies in Cohort 2

Figure 3

126x105mm (144 x 144 DPI)

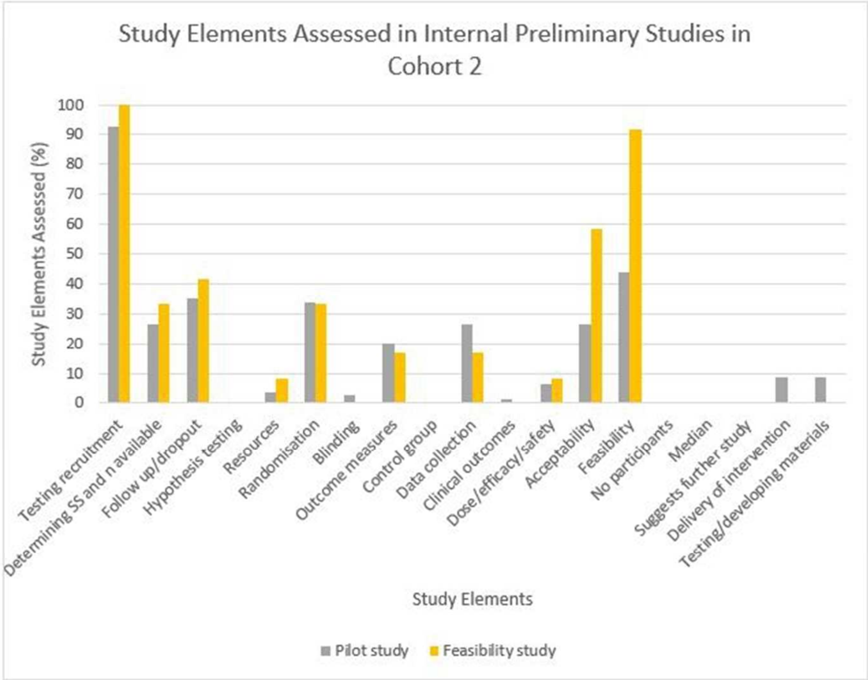

Figure 4: Study elements assessed in internal preliminary studies in Cohort 2

Figure 4

129x106mm (144 x 144 DPI)

BMJ Open

The role of feasibility and pilot studies in randomised controlled trials: a retrospective cohort study

Journal:	*BMJ Open*
Manuscript ID	bmjopen-2018-022233.R1
Article Type:	Research
Date Submitted by the Author:	12-Apr-2018
Complete List of Authors:	Blatch-Jones, Amanda; National Institute for Health Research Evaluation, Trials and Studies Coordinating Centre (NETSCC), University of Southampton<br>Pek, Wei; University of Southampton, Faculty of Medicine<br>Kirkpatrick, Emma ; University of Southampton, Southampton Clinical Trials Unit<br>Ashton-Key, Martin; University of Southampton, NETSCC
**Primary Subject Heading**:	Research methods
Secondary Subject Heading:	Evidence based practice
Keywords:	Randomised Controlled Trials, Pilot studies, Feasibility studies, Health Technology Assessment

SCHOLARONE™
Manuscripts

Title Page

The role of feasibility and pilot studies in randomised controlled trials: a retrospective cohort study

Amanda Blatch-Jones[a*]
Senior Research Fellow

Wei Pek[b]
Medical student, BM5 Medicine

Emma Kirkpatrick[c]
Trial Manager

Martin Ashton-Key[a]
Scientific Director and Consultant in Public Health Medicine

[a]National Institute for Health Research
Evaluation, Trials and Studies Coordinating Centre (NETSCC)
University of Southampton
Alpha House, Enterprise Road
Southampton SO16 7NS
UK

[b] Faculty of Medicine
University of Southampton
Southampton General Hospital
Southampton
UK SO16 6YD

[c] Southampton Clinical Trials Unit
University of Southampton
Southampton General Hospital
Southampton
UK SO16 6YD

*Corresponding author's email address: ajy5@soton.ac.uk

For peer review only

For peer review only - http://bmjopen.bmj.com/site/about/guidelines.xhtml

Abstract

Objectives: To assess the value of pilot and feasibility studies to randomised controlled trials (RCTs) funded by the National Institute for Health Research (NIHR) Health Technology Assessment (HTA) programme. To explore the methodological components of pilot / feasibility studies and how they inform full RCTs.

Study design: Retrospective cohort study.

Setting: Both cohorts included NIHR HTA Programme funded studies in the period 1 January 2010-31 December 2014 (decision date). Cohort 1: Standalone pilot/feasibility studies published in the HTA Journal or accepted for publication. Cohort 2: all funded RCT applications funded by the HTA Programme, including reference to an internal and/or external pilot/feasibility study. The methodological components were assessed using an adapted framework from a previous study.

Main outcome measures: The proportion of standalone pilot and feasibility studies which recommended proceeding to full trial and what study elements were assessed. The proportion of 'HTA funded' trials which used internal and external pilot and feasibility studies to inform the design of the trial.

Results: Cohort 1 identified 15 standalone pilot/feasibility studies. Study elements most commonly assessed were *testing recruitment* (100% in both groups), *feasibility* (83%, 100%) and *suggestions for further study/investigation* (83%, 100%). Cohort 2 identified 161 'HTA funded' applications: 59 cited an external pilot/feasibility study where *testing recruitment* (50%, 73%) and *feasibility* (42%, 73%) were the most commonly reported study elements: 92 reported an internal pilot/feasibility study where *testing recruitment* (93%, 100%) and *feasibility* (44%, 92%) were the most common study elements reported.

Conclusions: 'HTA funded' research which includes pilot and feasibility studies assesses a variety of study elements. Pilot and feasibility studies serve an important role when determining the most appropriate trial design. However, how they are reported and in what context, requires caution when interpreting the findings and delivering a definitive trial.

Keywords: Randomised Controlled Trials, Pilot studies, Feasibility studies, Health Technology Assessment

For peer review only

For peer review only - http://bmjopen.bmj.com/site/about/guidelines.xhtml

Article summary

Strengths and limitations of this study

- This paper assesses the role of pilot and feasibility studies funded by the NIHR HTA programme
- The study found that pilot and feasibility studies share common elements when contributing to the design of a trial
- The study contributes to the growing literature in this area and demonstrates the value of pilot and feasibility studies to the progression to full RCTs
- Although the data covers a five year period, the number of eligible studies are small and only report from one NIHR Programme

Acknowledgements: We would like to acknowledge the NIHR HTA Programme for providing data support during the preliminary stage of the study and the NETSCC Reporting Services team for identifying the relevant applications for the cohort. Professor Jane Blazeby for providing external expert advice during the study.

Ethic statement: This study did not require ethics approval as no patient or clinical data were required.

Funding statement: This study was supported by the NIHR Evaluation, Trials and Studies Coordinating Centre through the Research on Research programme as part of a University of Southampton, Faculty of Medicine, BM5 Medicine, 4th Year project. The views and opinions are those of the authors and do not necessarily reflect those of the Department of Health, or of NETSCC. This research received no specific grant from any funding agency in the public, commercial or not-for-profit sectors.

Contribution of Authors: The study was conceived and designed by MAK, ABJ and EK. The work was undertaken by WP as part of a 4th medical student research project, supervised and supported by MAK, ABJ and EK. Analysis and quality assurance were conducted by WP and MAK and the Access database was prepared by ABJ. All authors read and approved the final manuscript. ABJ is guarantor of the study.

Data sharing: The datasets used and analysed, and anonymised data are available on request from the corresponding author.

Licence for publication: The Corresponding Author has the right to grant on behalf of all authors and does grant on behalf of all authors, a worldwide license to the Publishers and its licensees in perpetuity, in all forms, formats and media (whether known now or created in the future), to i) publish, reproduce, distribute, display and store the Contribution, ii) translate the Contribution into other languages, create adaptations, reprints, include within collections and create summaries, extracts and/or, abstracts of the Contribution, iii) create any other derivative work(s) based on the Contribution, iv) to exploit all subsidiary rights in the Contribution, v) the inclusion of electronic links from the Contribution to third party material where-ever it may be located; and, vi) license any third party to do any or all of the above.

Competing interests: The authors have no competing financial interests; however, ABJ and MAK are employed by the University of Southampton to work for NETSCC. ABJ is employed as the Senior Research Fellow for the Research on Research programme and has worked for NETSCC (and its predecessor organisation) in various roles since 2008. MAK is the scientific

For peer review only - http://bmjopen.bmj.com/site/about/guidelines.xhtml

director at NETSCC and was an editor of the Health Technology Assessment journal. EK is employed by the University of Southampton and works at Southampton's Clinical Trials Unit. EK worked for the Research on Research programme during June 2014 to December 2015. WP was a 4th Year BM5 Medicine student at the University of Southampton.

Abstract word count: 299
Main text word count: 4388

For peer review only

INTRODUCTION

Pilot and feasibility studies have an important role to play in the development of Randomised Controlled Trials (RCTs). If appropriately used, pilot and feasibility studies can provide sufficient methodological evidence about the design planning and justification of a trial. They are often undertaken to inform elements of the main trial design, but they can also be used to reduce or eliminate problems that limit the successful delivery of trials. In 2009, the Lancet published a paper that highlighted the extent to which research is wasted, and that loss is as much as 85% of research investment.[1] Given the cost and time of investment from researchers and major health research funders, there is now a growing demand to assess and examine where improvements need to be made to the design and conduct of trials.[2] Poorly designed trials could include non-reference to a pre-existing systematic literature review or bias generated by inadequate concealment of treatment allocation.[1] Research by Cooper *et al.* has also shown variability between external pilots and the prediction for randomisation and attrition rates.[3] As a result, much attention has primarily focused on the design, conduct and analysis of clinical research to determine where improvements are needed to reduce waste in research.

Over the last ten years, we have seen how pilot and feasibility studies have become an important feature in terms of gathering evidence to inform the development of a full trial. There is now an extension to the Consolidated Standards of Reporting Trials (CONSORT) which provides guidance for pilot and feasibility studies being conducted prior to a main trial.[4] Conducting a pilot or feasibility study to determine any uncertainties prior to the main trial may help to eradicate issues and thus inform the definitive trial. More importantly perhaps, is the role pilot and feasibility studies can have in modifying the design and conduct, and therefore increasing the value of the research, helping to avoid methodological design flaws and reducing the burden of research waste.

Despite the growing importance of pilot and feasibility studies there is still a lack of clarity about the use of the two terms.[5-7] In 2008, the Medical Research Council (MRC) published guidance on developing and evaluating complex interventions to demonstrate the value and importance of pilot and feasibility studies as a key element in the development and evaluation process. However, the guidance did not attempt to explain or provide any definition for the terms 'pilot' and 'feasibility'.[7] It was not until two years later that Thabane *et al.* reviewed the key aspects of pilot studies and provided a detailed account of pilot studies which included a number of definitions.[6] Around the same time (2009), the National Institute for Health Research, Evaluation, Trials and Studies Coordinating Centre (NETSCC) published a support document detailing what feasibility and pilot studies are.

- Feasibility studies are defined as "pieces of research done before a main study in order to answer the question "Can this study be done?". They are used to estimate important parameters that are needed to design the main study…feasibility studies do not evaluate the outcome of interest."
- Pilot studies are defined as "a version of the main study that is run in miniature to test whether the components of the main study can all work together. It is focused on the processes of the main study…it will therefore resemble the main study in many respects."[8]

These definitions have gone some way to aid the understanding of when it is appropriate to do pilot or feasibility studies as part of the definitive trial. These definitions are now widely used across NIHR.

Despite the importance of the role of pilot and feasibility studies in informing RCTs, there is little empirical evidence about the use of these studies in informing future trials. For example, the Lancet series in 2014 did not make reference to the usefulness of pilot and feasibility studies in

For peer review only - http://bmjopen.bmj.com/site/about/guidelines.xhtml

the context of increasing value and reducing waste in research design, conduct or analysis.[9] Lancaster *et al.* and Arain *et al.* provided a methodological framework to assess how pilot studies are used to inform the conduct and reporting of pilot studies.[5 10] Both described the challenges and complexities in the reporting of pilot studies. Arain *et al.* further explored these complexities in relation to feasibility studies and full trials. More recently, research has begun to explore the differences between internal and external pilot studies and their contribution to main trials, and the appropriateness of pilot and feasibility studies for estimating the sample size.[3 11 12]

The aim of this study is to contribute evidence to this important gap in the current literature. The objective of this paper is to describe the process and results of how, and in what way, pilot and feasibility studies have been used to inform full RCTs.

For peer review only

For peer review only - http://bmjopen.bmj.com/site/about/guidelines.xhtml

METHODS

The Health Technology Assessment (HTA) Programme has a long history of commissioning pilot and feasibility studies. Therefore, the published reports (NIHR HTA Journal) of standalone pilot and feasibility studies were examined to determine which elements of research design are most often assessed. Applications for funded HTA trials were also assessed to establish how full trials were informed by previously completed pilot / feasibility studies as well as pilot studies embedded within the trial.

Data source

An assessment of the NIHR HTA Programme over a five-year period (2010-2014) was conducted using two retrospective cohorts. There were two cohorts due to the data being homogenous (data for cohort 1 was taken from the published HTA journal article and data for cohort 2 was taken from the HTA application form).

In order to identify the included studies for both cohorts we
1. Reviewed the project title in the application form and the journal article title
2. Reviewed the abstract / executive summary
3. Reviewed the full Journal article or HTA application form

Sample selection

Cohort 1: Standalone pilot and feasibility studies

Standalone pilot / feasibility studies funded by the HTA Programme with a fund decision date from 01 January 2010 to 31 December 2014, which have published in the HTA Journal or are currently being prepared for publication and have been signed off by the editors (only those in production) were included. The published Journal/approved final version of the published report was used as the source for data extraction. The standalone studies were categorised into 'pilot study', 'feasibility study' or 'both'.

Cohort 2: Randomised Controlled Trials

Trials funded through the HTA Programme with a fund decision date between 01 January 2010 to 31 December 2014 were included. The application form of a funded trial was used as the source for data extraction. The trials were categorised based on the type of pilot and/or feasibility: 'external / previous pilot study', 'external / previous feasibility study', 'internal pilot study', 'internal feasibility study' or 'other (mixed study)'.

NIHR Evaluation, Trials and Studies Management Information System (NETS MIS) was used to identify the two cohorts and extract the relevant documents needed for data extraction. Search terms were used to search for relevant data to ensure the feasibility of future replications. The key search terms used were: pilot, feasibility, preliminary work, earlier/previous study.

In addition to the search using key terms, a targeted search was carried out on specific areas of the application form. Focusing on specific areas of the application was relevant in identifying where the elements of the study design would most likely be described in relation to the pilot and/or feasibility study.

Piloting

Data extraction tables for cohort 1 and cohort 2 were piloted with an initial sample of 10 studies. No changes were required to the classification system previously adopted by Arain *et al.* as a result of the pilot work.

For peer review only - http://bmjopen.bmj.com/site/about/guidelines.xhtml

Classification systems
The definition of pilot and feasibility studies agreed by NIHR for four Programmes was used for the purpose of this study[8]. These definitions were also used by Arain *et al.*[10]

The classification systems developed by Arain *et al.* and Bugge *et al.* were adapted to determine what elements of a study design were assessed or used to inform the full trial (see *Table 1*).[10 13] In both cohorts, the elements of the study design were examined in terms of

a. Did the study explicitly state it assessed any of these elements? (yes/no)
b. Were there any recommended changes as a result of the assessment? A yes response was defined as: the authors reported a change / recommendation to be considered but did not necessarily report what that change was. If the authors did not explicitly state a recommendation, it was assumed that no changes were required.

The text pertaining to the pilot and/or feasibility study was also extracted for quality assurance purposes.

Insert Table 1 here

Two additional study elements were included in cohort two which were not reported in cohort one. These were '*delivery of intervention*' and '*testing/developing materials*'.

Patient and Public Involvement
There was no patient or public involvement in the design of the study due to the nature of the project (part of a University of Southampton, Faculty of Medicine, BM5 Medicine, 4th Year project). There was no participant recruitment involved in the project, as all data were taken from the published article or the HTA application.

Data quality and assurance
Our approach to quality assurance was guided by the Strengthening the Reporting of Observational Studies in Epidemiology (STROBE), which although designed for observational studies, could be applied to the processes we used in this current study.

For both cohorts, WP extracted all data and a second person assessed and reviewed the data to ensure the accuracy of data extraction. All of cohort 1 was assessed followed by 15% of cohort 2 (purposive sampling of 5% of the cohort followed by 10% randomly selected application forms). The remaining 85% was subsequently reviewed by MAK to determine the reliability and validity of the data extraction and usability of the adapted template. All disagreements were discussed by the team and were resolved by consensus. Data management was undertaken by WP with support from ABJ.

Data analysis
Data for each study, based on the framework developed by Arain *et al.* (see Table 1), was captured using Microsoft Access 2010 (Microsoft Corporation, Redmond, WA, USA).[10] The study design elements were entered onto an Access form and where a study element was reported a 'yes' response was captured. A separate Access form was developed for each included study for both cohorts. Both cohorts were exported into Microsoft Excel 2010 (Microsoft Corporation, Redmond, WA, USA) and then subsequently into Statistical Product and Service Solutions (SPSS) version 22 (IMB Corporation, Armonk, NY, USA). Excel was used to calculate the median and the range for cohort 1 only. Data were analysed and interpreted using descriptive statistics to determine the frequency of the study design elements and how often changes were recommended for full trials.

For peer review only - http://bmjopen.bmj.com/site/about/guidelines.xhtml

Results

In cohort 1 we identified 47 published standalone pilot and/or feasibility studies and in cohort 2 we identified 303 'HTA funded' RCTs during the five-year period (01 January 2010 – 31 December 2014). Fifteen standalone studies were identified as eligible for cohort 1 and 161 funded HTA applications were identified and eligible for cohort 2.

Cohort 1

A total of 47 standalone studies were identified. Thirty-two were excluded on further examination due to not being a pilot or feasibility study (we did not categorise the excluded studies by study design). The remaining 15 studies were categorised into three separate groups (see *Figure 1*). We found that 13 of the 15 study elements included in the adapted framework were assessed in standalone pilot studies compared to nine study elements in feasibility studies.

In this cohort, it was found that seven studies used the terms "pilot" and "feasibility" interchangeably and it was difficult to determine, even with the NIHR definition, what type of study was undertaken. Therefore, it was not possible to accurately determine which study elements belonged to which, and in some cases the authors described the conduct of both pilot and feasibility work. The team agreed to combine pilot and feasibility together in this instance, which was also found in Arain *et al*.

Insert Figure 1 here

The median number of participants for the standalone studies (n=15) was 46. Of the 15 eligible standalone pilot and/or feasibility studies, the most commonly reported study design element was *testing recruitment*. In all three groups (pilot studies, feasibility studies and pilot/feasibility studies), all 15 studies assessed recruitment (6/6, 2/2 and 7/7 respectively) (see *Table 2*). Half of these also reported recommended changes to recruitment (3/6, 1/2 and 3/7 respectively). Interestingly, both feasibility studies only (2/2) and pilot/feasibility groups (7/7) assessed the need for *further study* and suggested recommended changes (*further study* referred to whether further investigation was required using a large RCT and where future trial data could be of benefit).

Insert Table 2 here

Cohort 2

A total of 303 'HTA funded' applications were identified. Eighty-two were excluded upon examination as they were not RCTs (for example cohort studies, diagnostic accuracy test studies and we did not categorise the excluded studies by study design) and a further 60 applications were excluded due to not being informed by any external or internal pilot and/or feasibility study. The remaining 161 applications were reviewed and subsequently grouped into five categories (see *Figure 2*).

1. External pilot studies (n=48)
2. External feasibility studies (n=11)
3. Internal pilot studies (n=80)
4. Internal feasibility studies (n=12)
5. Other (n=10)

As the HTA application was used as the source of data extraction, the outcome of the internal pilot/feasibility study was not available (n=92). For the 59 applications where an external pilot / feasibility study was referenced, we found that not all of these studies provided information relating to the number of participants that took part in the pilot/feasibility study. We did not go

For peer review only - http://bmjopen.bmj.com/site/about/guidelines.xhtml

back to the original journal article to retrieve this information. Therefore, it was not appropriate to estimate the median or inter quartile range for this cohort.

The *others* group comprised applications that were informed by a combination of more than one preliminary study (e.g. internal and/or external pilot study and/or feasibility study). Of those 10 applications,

- Six of the ten were informed by external pilot studies,
- Seven of the ten were informed by external feasibility studies,
- Seven of the ten were informed by internal pilot studies and
- One of the ten was informed by an internal feasibility study.

No further analysis was conducted on these 10 applications due to the diverse nature of the study types in this subgroup.

Insert Figure 2 here

External pilot and feasibility studies

Of the 161 applications, 29.8% (48/161) reported or cited a previous external pilot study not recently done by the applicant and 6.8% (11/161) reported an external feasibility study. For this subset, all of the study elements (n=17) were assessed by external pilot studies but no single study assessed all 17 elements. By comparison, 13 of the 17 study elements were assessed by external feasibility studies (see *Table 3*).

In terms of the study elements, *testing recruitment*, *determining the sample size and numbers available*, and the *feasibility* were the most commonly reported in both external pilot and feasibility studies. The number of reported recommended changes based on the results of the external pilot or feasibility study were however minimal. Although, in some applications it was possible to detect a change, the authors did not explicitly state a recommended change. Therefore, it was not possible to determine whether this was based on the pilot or feasibility study, or some other factor.

Insert Table 3 here

Internal pilot and feasibility studies

Of the 161 applications, 49.7% (80/161) reported an internal pilot study and 7.5% (12/161) reported an internal feasibility study. Due to the source of data extraction (the application form) it was not possible to determine whether the funded internal pilot or feasibility study had made any recommended changes, as the internal study had not yet been conducted.

For the internal studies, we found 14 of the 17 study elements were being assessed by internal pilot studies compared to 10 study elements in feasibility studies. Based on assessment only, the most common study element to be reported was *testing recruitment* (74/80 and 12/12 respectively) and *feasibility* (35/80 and 11/12 respectively) for both internal pilot and feasibility study (see *Table 4*). There were several similarities between a number of study elements assessed by both pilot and feasibility studies.

Insert Table 4 here

For peer review only - http://bmjopen.bmj.com/site/about/guidelines.xhtml

For peer review only

Discussion

This study found that pilot and feasibility studies do play a role in the development and design of definitive RCTs. In both cohorts, it was clear that two study elements were commonly assessed: t*esting recruitment* and *feasibility*. This has important implications for the success of a trial, given that many trials struggle with recruitment and often request extensions or become at risk of closure.[14 15] Our findings showed how trials use pilot and/or feasibility studies in an attempt to assess and evaluate prior to a full trial, whether it is likely to be able to recruit its target sample size and whether the study is indeed feasible as a full trial. In both cohorts, we found pilot studies assessed more study elements than feasibility studies. This also applied to the internal and external study groups in cohort 2; external and internal pilot studies were used to assess more study elements than feasibility studies.

Strengths and weaknesses of the study and in relation to other studies

The main strength of the study was the inclusion of all 'HTA funded' studies over a five-year period. Although cohort one (standalone) only included 15 studies, this was as expected. For this cohort, we identified an increase in almost all of the study elements being assessed compared to earlier work by Arain *et al.*[10] In cohort two, over half of the 'HTA funded' applications included a pilot and/or feasibility study (internal and/or external) (161/303). Compared to Arain *et al.*[10] the findings were similar for the external pilot and feasibility studies cited in terms of the number of study elements assessed and the number of studies included. For example, *testing recruitment* was the most frequently reported element for pilot studies in both the current study and Arain *et al.*, and d*etermining the sample size and the numbers available* was identical in both studies. However, *randomisation*, *clinical outcomes* and *feasibility* were reported more frequently by Arain *et al.* than the current study. For the external feasibility studies, the current study found more study elements being assessed than that of Arian *et al.* in terms of *testing recruitment*, *determining the sample size and the numbers available*, *randomisation*, *acceptability*, *feasibility* and *follow up/drop out*.

For the internal pilot studies, similar findings were found when comparing Arain *et al.* to the current study: d*etermining the sample size and the numbers available, randomisation* and *clinical outcomes* were assessed more in Arain *et al.* than the current study. As with the internal feasibility studies, we found the current study to report more study elements being assessed than that of Arain *et al.*: *testing recruitment*, *determining the sample size and the numbers available*, *follow up/drop out*, *randomisation*, *acceptability* and *feasibility*. These differences, particularly found with the feasibility studies could be associated with changes over time in the use and understanding of feasibility studies.

This study relied on an adapted version of the Arain *et al.* framework. As some of the study elements were expanded and new ones were added a direct comparison with Arain *et al.* findings is limited.[10] Given the subjective nature of some of the study elements, we chose to quality assure all data to eradicate and reduce any known errors. Since the analysis was based explicitly on the reporting of the applicants, and did not include any subjective account or interpretation of what was reported, we may have under reported the number of study elements assessed and/or recommended.

We also noted a mismatch in numbers between those assessing study elements and those where recommendations were made in cohort one. This was due, in part, to how each study element was reported by the applicants. For example if a study did not specify that they had assessed these elements but made recommendation for changes, we only inferred that they assessed it, but it could not be recorded in the data, hence the mismatch in the findings. This

For peer review only - http://bmjopen.bmj.com/site/about/guidelines.xhtml

does however highlight the importance of clearly reporting how, what and where the pilot and/or feasibility study had an impact on the design of the definitive trial.

Implications

The level of appropriateness in the reporting of pilot and feasibility studies could largely be affected by the lack of clarity and awareness of the different study requirements. Despite the growing literature on improving the quality of research to reduce waste in research, there is limited literature pertaining to how pilot and feasibility studies fit into this agenda for change. From what literature there is on pilot and feasibility studies, there is still some confusion about when, why and how it is appropriate to conduct a pilot and/or feasibility study. The findings in this study, even with the use of a well-defined definition by NIHR, still found evidence where applicants did not adhere to the HTA definitions for "pilot" and "feasibility" study on research applications. The terminology is still being used interchangeably. Although the commentary on pilot studies by Thabane *et al.* gives a detailed account of the appropriateness of why and how to conduct a pilot study, a comparison with feasibility studies is lacking.[6] It would be helpful to have a more formal distinction between these two terminologies as suggested by Arain *et al.* A recent study by Eldridge *et al.* goes some way to rectify this by developing a conceptual framework for defining pilot and feasibility studies.[16] The conceptual framework shows promising results, by being compatible with the MRC guidance on complex interventions,[7] and their descriptor of pilot studies is similar to that of the NIHR (NIHR definition). However, it is important to note that the Eldridge *et al.* conceptual framework is slightly different from that adopted by the NIHR.[16] The clear lack of dichotomy between pilot and feasibility studies is an area for future consideration, not only for funders to encourage more conformity to the published definitions, but for researchers to make better use of the existing literature to better understand the distinction between pilot and feasibility studies.

Having clear definitions of when to use pilot and feasibility studies is clearly important for confirming the progression to a full trial. However, it is also imperative to note the limitations of pilot and feasibility studies and when it is not appropriate to conduct this type of study. Pilot and feasibility studies are to assist and direct the design stage of a trial, they should not be used to assess effectiveness, make claims about whether the treatment works or not, or perform sample size calculations to produce effect size estimates for a larger trial. Feasibility studies are not adequately powered to assess effectiveness; the sample sizes are too small to give true effect size estimates. Although, research focusing on pilot studies and the contribution to sample sizes, randomisation and attribution rates provide promising results, the findings are not applicable to all types of pilot and feasibility studies (internal, external) and requires minimal or no change to the full trial.[3 11 12] So how generalisable these findings are should be used with caution. Both pilot and feasibility studies have a role to play and are extremely important to the design stage of a trial. However, how they are reported and in what context, requires caution especially when interpreting the findings and delivering a definitive trial.[17]

Conclusion and recommendations

'HTA funded' research which is inclusive of pilot and feasibility studies is very likely to assess a variety of study elements, which have been evidence-based through this current study using an adapted version of Arain *et al.* framework.[10] However, not reviewing the impact of the preliminary work once the trial commences, we have no way of knowing whether the pilot and/or feasibility studies recommendations were instrumental in the successful completion of the trial. If

For peer review only - http://bmjopen.bmj.com/site/about/guidelines.xhtml

we are able to demonstrate the value of pilot and feasibility studies we need to place greater emphasis on not only their role in the design stage of a trial but also how this preliminary work contributed favourably, or not, to the completion of the definitive trial. The internal pilot and/or feasibility studies reported in cohort 2 could be used for the basis of continual work in this area. By following up on this cohort we would be able to analyse the successful delivery of the definitive trial and whether the preliminary work had any bearing on this success.

Recommendations include a larger sample of studies across other UK health research funding agencies to determine the frequency and importance of those study elements reported here. A further assessment between the study elements noted in the pilot and feasibility studies and how this impacted on the eventual design and conduct of the definitive trial would certainly add value. This could be achieved by prospectively evaluating the ongoing use of pilot and feasibility studies in cohort two (specifically the internal pilot and/or feasibility studies) as well as future funded applications to the HTA programme. Highlighting the need for better reporting of pilot and feasibility studies should be regarded as relevant to all research funding bodies. And as such, better guidelines for the design, conduct, analysis and reporting of pilot and feasibility studies are still needed.

Future work could therefore include widening the study outcomes presented here to other NIHR funded programmes, for example Programme Grants for Applied Research (PGfAR). Funders might want to consider the use of Arain *et al.* framework when considering the funding of pilot / feasibility studies. Where appropriate this could contribute to maximising the benefit of research and reducing the extent to which research is wasted. If we find ways to appropriately address the flaws detected at the design and conduct stages of research, then we could start to see how research adds value and reduces the amount of research waste. In order to achieve this, we need clearly defined terminology which is inclusive of funding agencies and researchers' perspective; empirical evidence on the reporting and appropriate use of pilot and feasibility studies, in terms of favourable study elements and; an evaluation of the contribution to definitive trial outcomes.

For peer review only - http://bmjopen.bmj.com/site/about/guidelines.xhtml

Table 1: Elements of a study design adapted from Arain *et al.*

**The methodological components included as reported by and included from Arain et al:**
**Methods related**
Testing Recruitment
Determining the sample size / numbers available
Follow up / dropout
Hypothesis testing
Resources
Randomisation
Blinding
Outcome measures
Control group
Data collection
Further study suggested
**Intervention related**
Dose / efficacy / safety
Clinical outcomes
Acceptability
Feasibility
In addition to the above, Cohort 2 included:
Delivery of the intervention
Testing/developing materials

For peer review only

For peer review only - http://bmjopen.bmj.com/site/about/guidelines.xhtml

Table 2: Cohort1 - Study elements captured in pilot studies, feasibility studies and pilot/feasibility studies

	Pilot studies only (n=6)			Feasibility studies only (n=2)			Pilot/Feasibility studies (n=7)		
**Study elements**	**Assessed (A): Number (%)**	**Recommended changes (RC): Number (%)**	**A and RC: Number (%)**	**Assessed (A): Number (%)**	**Recommended changes (RC): Number (%)**	**A and RC: Number (%)**	**Assessed (A): Number (%)**	**Recommended changes (RC): Number (%)**	**A and RC: Number (%)**
Testing recruitment	6 (100.0)	3 (50.0)	3 (50.0)	2 (100.0)	1 (50.0)	1 (50.0)	7 (100.0)	3 (42.9)	3 (42.9)
Determining Sample Size and/or number available	5 (83.3)	1 (16.6)	0	1 (50.0)	2 (100.0)	1 (50.0)	5 (71.4)	1 (14.3)	1 (14.3)
Follow up/dropout	4 (66.6)	3 (50.0)	3 (50.0)	0	1 (50.0)	0	5 (71.4)	2 (28.6)	1 (14.3)
Hypothesis testing	2 (33.3)	0	0	1 (50.0)	0	0	1 (14.3)	0	0
Resources	4 (66.6)	3 (50.0)	2 (33.3)	2 (100.0)	0	0	5 (71.4)	1 (14.3)	1 (14.3)
Randomisation	4 (66.6)	0	0	0	0	0	6 (85.7)	1 (14.3)	1(14.3)
Blinding	0	0	0	0	0	0	2 (28.6)	0	0
Outcome measures	5 (83.3)	4 (66.6)	4 (66.6)	2 (100.0)	1 (50.0)	1 (50.0)	6 (85.7)	4 (57.1)	3 (42.9)
Control group	0	0	0	0	0	0	1 (14.3)	0	0
Data collection	3 (50.0)	0	0	0	1 (50.0)	0	3 (42.9)	1 (14.3)	0
Clinical outcomes	3 (50.0)	2 (33.3)	1 (16.6)	1 (50.0)	0	0	3 (42.9)	0	0
Dose/efficacy/ safety	2 (33.3)	1 (16.6)	1 (16.6)	0	0	0	2 (28.6)	0	0
Acceptability	4 (66.6)	0	0	1 (50.0)	0	0	6 (85.7)	0	0
Feasibility	5 (83.3)	0	0	2 (100.0)	0	0	7 (100.0)	0	0
Suggests further study	5 (83.3)	4 (66.6)	4 (66.6)	2 (100.0)	2 (100.0)	2 (100.0)	7 (100.0)	7 (100.0)	7 (100.0)
Median number of participants (IQR) [Range]	47.5 (39.25-85) [21-99]			14 (7-21) [0-28]			58 (35.5-173) [29-313]		

For peer review only - http://bmjopen.bmj.com/site/about/guidelines.xhtml

Table 3: Cohort 2 - Study elements captured in external pilot and feasibility studies

Study elements	External pilot study (n=48)		External feasibility study (n=11)	
	Assessed Number (%)	Recommended changes Number (%)	Assessed Number (%)	Recommended changes (n)
Testing recruitment	24 (50.0)	3 (6.3)	8 (72.7)	0
Determining sample size and/or number available	24 (50.0)	1 (2.1)	4 (36.4)	1 (9.1)
Follow up/dropout	16 (33.3)	0	3 (27.3)	0
Hypothesis testing	10 (20.8)	0	2 (18.2)	0
Resources	2 (4.2)	0	1 (9)	0
Randomisation	7 (14.6)	0	3 (27.3)	0
Blinding	4 (8.3)	1 (2.1)	0	0
Outcome measures	10 (20.8)	1 (2.1)	1 (9.1)	0
Control group	3 (6.3)	0	0	0
Data collection	6 (12.5)	0	2 (18.2)	0
Clinical outcomes	12 (25.0)	0	1 (9.1)	0
Dose/efficacy/safety	14(29.2)	1 (2.1)	0	0
Acceptability	17 (35.4)	0	4 (36.4)	0
Feasibility	20 (41.7)	0	8 (72.7)	0
Suggests further study	8 (16.6)	1 (2.1)	1 (9.1)	0
Delivery of intervention	8 (16.6)	2 (4.2)	0	0
Testing/developing materials	3 (6.3)	0	1 (9.1)	0

Table 4: Cohort 2 - Study elements captured in internal pilot and feasibility studies

Study elements	Internal pilot study (n=80)	Internal feasibility study (n=12)
	Assessed No. (%)	Assessed No. (%)
Testing recruitment	74 (92.5)	12 (100.0)
Determining sample size and/or number available	21 (26.3)	4 (33.3)
Follow up/dropout	28 (35.0)	5 (41.7)
Hypothesis testing	0	0
Resources	3 (3.8)	1 (8.3)
Randomisation	27 (33.8)	4 (33.3)
Blinding	2 (2.5)	0
Outcome measures	16 (20.0)	2 (16.7)
Control group	0	0
Data collection	21 (26.3)	2 (16.7)
Clinical outcomes	1 (1.3)	0
Dose/efficacy/safety	5 (6.3)	1 (8.3)
Acceptability	21 (26.3)	7 (58.3)
Feasibility	35 (43.8)	11 (91.7)
Suggests further study	0	0
Delivery of intervention	7 (8.8)	0
Testing/developing materials	7 (8.8)	0

For peer review only - http://bmjopen.bmj.com/site/about/guidelines.xhtml

References

1. Chalmers I, Glasziou P. Avoidable waste in the production and reporting of research evidence. *The Lancet* 2009;374(9683):86-89. doi: doi: DOI: 10.1016/S0140-6736(09)60329-9
2. Moher D, Glasziou P, Chalmers I, et al. Increasing value and reducing waste in biomedical research: who's listening? *The Lancet* 2016;387(10027):1573-86. doi: https://doi.org/10.1016/S0140-6736(15)00307-4
3. Cooper CL, Whitehead A, Pottrill E, et al. Are pilot trials useful for predicting randomisation and attrition rates in definitive studies: A review of publicly funded trials. *Clinical Trials* 2018:1740774517752113.
4. Eldridge SM, Chan CL, Campbell MJ, et al. CONSORT 2010 statement: extension to randomised pilot and feasibility trials. *BMJ* 2016;2(1):64.
5. Lancaster GA, Dodd S, Williamson PR. Design and analysis of pilot studies: recommendations for good practice. *Journal of Evaluation in Clinical Practice* 2004;10(2):307-12. doi: 10.1111/j..2002.384.doc.x
6. Thabane L, Ma J, Chu R, et al. A tutorial on pilot studies: the what, why and how. *BMC medical research methodology* 2010;10(1):1.
7. Craig P, Dieppe P, Macintyre S, et al. Developing and evaluating complex interventions: The new Medical Research Council guidance. *International Journal of Nursing Studies* 2013;50(5):587-92. doi: 10.1016/j.ijnurstu.2012.09.010
8. NIHR. Feasibility and Pilot studies https://www.nihr.ac.uk/funding-and-support/documents/funding-for-research-studies/research-programmes/PGfAR/Feasibility%20and%20Pilot%20studies.pdf 2018 [accessed 6 Feb 2018.
9. Ioannidis JP, Greenland S, Hlatky MA, et al. Increasing value and reducing waste in research design, conduct, and analysis. *Lancet* 2014;383(9912):166-75. doi: 10.1016/S0140-6736(13)62227-8
10. Arain M, Campbell MJ, Cooper CL, et al. What is a pilot or feasibility study? A review of current practice and editorial policy. *BMC medical research methodology* 2010;10:67. doi: 10.1186/1471-2288-10-67
11. Whitehead AL, Julious SA, Cooper CL, et al. Estimating the sample size for a pilot randomised trial to minimise the overall trial sample size for the external pilot and main trial for a continuous outcome variable. *Statistical methods in medical research* 2016;25(3):1057-73.
12. Billingham SA, Whitehead AL, Julious SA. An audit of sample sizes for pilot and feasibility trials being undertaken in the United Kingdom registered in the United Kingdom Clinical Research Network database. *BMC medical research methodology* 2013;13(1):104.
13. Bugge C, Williams B, Hagen S, et al. A process for Decision-making after Pilot and feasibility Trials (ADePT): development following a feasibility study of a complex intervention for pelvic organ prolapse. *Trials* 2013;14:353. doi: 10.1186/1745-6215-14-353
14. Raftery J, Young A, Stanton L, et al. Clinical trial metadata: defining and extracting metadata on the design, conduct, results and costs of 125 randomised clinical trials funded by the National Institute for Health Research Health Technology Assessment programme. *Health Technology Assessment* 2015;19(11):1-166.
15. Bower P, Brueton V, Gamble C, et al. Interventions to improve recruitment and retention in clinical trials: a survey and workshop to assess current practice and future priorities. *Trials* 2014;15(1):399.
16. Eldridge SM, Lancaster GA, Campbell MJ, et al. Defining feasibility and pilot studies in preparation for randomised controlled trials: development of a conceptual framework. *PLoS One* 2016;11(3):e0150205.

For peer review only - http://bmjopen.bmj.com/site/about/guidelines.xhtml

17. Vogel S, Draper-Rodi J. The importance of pilot studies, how to write them and what they mean. *International Journal of Osteopathic Medicine* 2017;23:2-3. doi: 10.1016/j.ijosm.2017.02.001

For peer review only

For peer review only - http://bmjopen.bmj.com/site/about/guidelines.xhtml

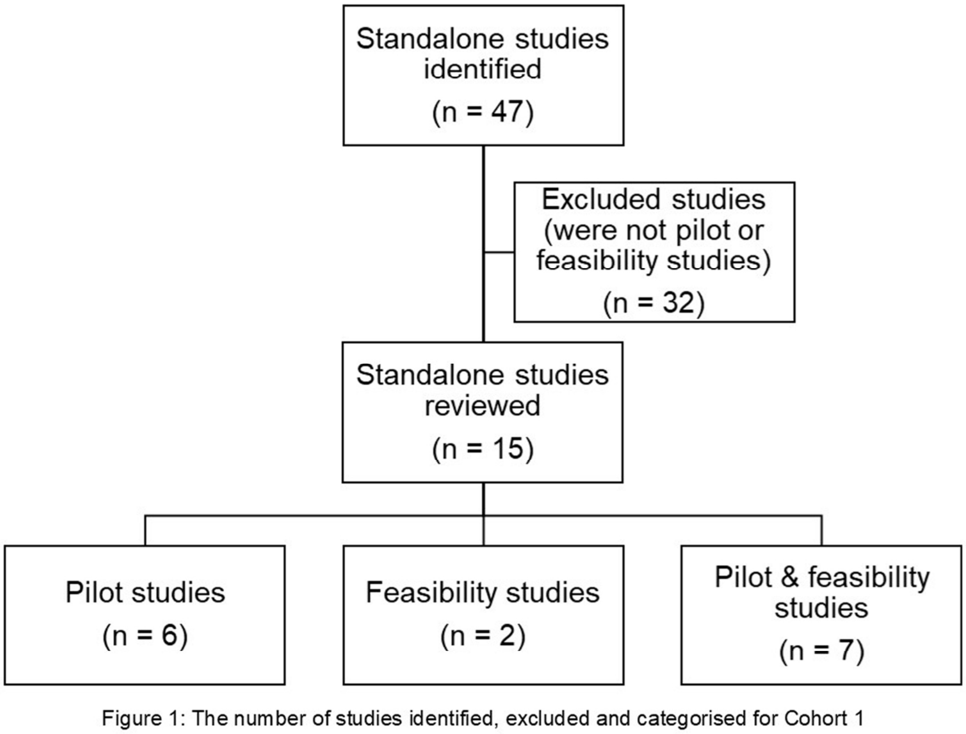

Figure 1: The number of studies identified, excluded and categorised for Cohort 1

165x126mm (300 x 300 DPI)

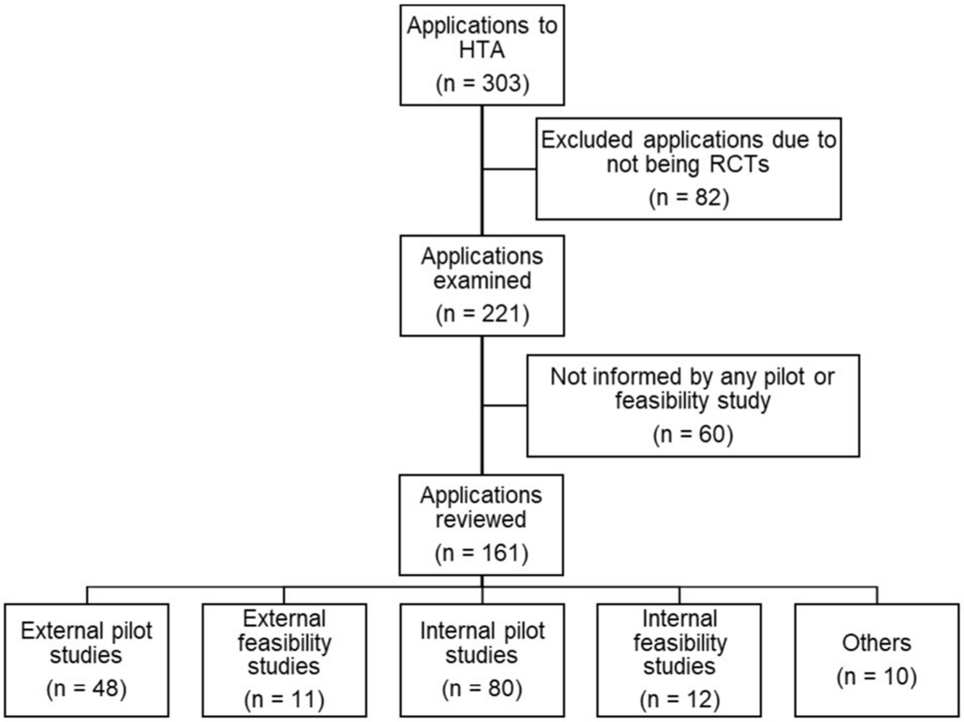

Figure 2: Flow chart showing the number of HTA funded applications for Cohort 2

Figure 2: Flow chart showing the number of HTA funded applications for Cohort 2

147x116mm (300 x 300 DPI)

STROBE Statement—Checklist of items that should be included in reports of ***cohort studies***

	Item No	Recommendation	Page No.
**Title and abstract**	1	(*a*) Indicate the study's design with a commonly used term in the title or the abstract	1
		(*b*) Provide in the abstract an informative and balanced summary of what was done and what was found	2
**Introduction**			
Background/rationale	2	Explain the scientific background and rationale for the investigation being reported	5
Objectives	3	State specific objectives, including any prespecified hypotheses	6
**Methods**			
Study design	4	Present key elements of study design early in the paper	7
Setting	5	Describe the setting, locations, and relevant dates, including periods of recruitment, exposure, follow-up, and data collection	7
Participants	6	(*a*) Give the eligibility criteria, and the sources and methods of selection of participants. Describe methods of follow-up	7
		(*b*) For matched studies, give matching criteria and number of exposed and unexposed	NA
Variables	7	Clearly define all outcomes, exposures, predictors, potential confounders, and effect modifiers. Give diagnostic criteria, if applicable	NA
Data sources/ measurement	8*	For each variable of interest, give sources of data and details of methods of assessment (measurement). Describe comparability of assessment methods if there is more than one group	7,8
Bias	9	Describe any efforts to address potential sources of bias	NA
Study size	10	Explain how the study size was arrived at	7
Quantitative variables	11	Explain how quantitative variables were handled in the analyses. If applicable, describe which groupings were chosen and why	NA
Statistical methods	12	(*a*) Describe all statistical methods, including those used to control for confounding	NA
		(*b*) Describe any methods used to examine subgroups and interactions	NA
		(*c*) Explain how missing data were addressed	NA
		(*d*) If applicable, explain how loss to follow-up was addressed	NA
		(*e*) Describe any sensitivity analyses	NA
**Results**			
Participants	13*	(a) Report numbers of individuals at each stage of study—eg numbers potentially eligible, examined for eligibility, confirmed eligible, included in the study, completing follow-up, and analysed	9,10, Fig1, Fig2
		(b) Give reasons for non-participation at each stage	9,10, Fig1, Fig2
		(c) Consider use of a flow diagram	Fig1, Fig2
Descriptive data	14*	(a) Give characteristics of study participants (eg demographic, clinical, social) and information on exposures and potential confounders	9,10
		(b) Indicate number of participants with missing data for each variable of interest	NA
		(c) Summarise follow-up time (eg, average and total amount)	NA
Outcome data	15*	Report numbers of outcome events or summary measures over time	NA

For peer review only

Main results	16	(*a*) Give unadjusted estimates and, if applicable, confounder-adjusted estimates and their precision (eg, 95% confidence interval). Make clear which confounders were adjusted for and why they were included	NA
		(*b*) Report category boundaries when continuous variables were categorized	NA
		(*c*) If relevant, consider translating estimates of relative risk into absolute risk for a meaningful time period	NA
Other analyses	17	Report other analyses done—eg analyses of subgroups and interactions, and sensitivity analyses	NA
**Discussion**			
Key results	18	Summarise key results with reference to study objectives	11
Limitations	19	Discuss limitations of the study, taking into account sources of potential bias or imprecision. Discuss both direction and magnitude of any potential bias	11
Interpretation	20	Give a cautious overall interpretation of results considering objectives, limitations, multiplicity of analyses, results from similar studies, and other relevant evidence	11
Generalisability	21	Discuss the generalisability (external validity) of the study results	11-13
**Other information**			
Funding	22	Give the source of funding and the role of the funders for the present study and, if applicable, for the original study on which the present article is based	3

*Give information separately for exposed and unexposed groups.

Note: An Explanation and Elaboration article discusses each checklist item and gives methodological background and published examples of transparent reporting. The STROBE checklist is best used in conjunction with this article (freely available on the Web sites of PLoS Medicine at http://www.plosmedicine.org/, Annals of Internal Medicine at http://www.annals.org/, and Epidemiology at http://www.epidem.com/). Information on the STROBE Initiative is available at http://www.strobe-statement.org.

For peer review only

BMJ Open

The role of feasibility and pilot studies in randomised controlled trials: a cross-sectional study

Journal:	*BMJ Open*
Manuscript ID	bmjopen-2018-022233.R2
Article Type:	Research
Date Submitted by the Author:	22-Jun-2018

Complete List of Authors:	Blatch-Jones, Amanda; National Institute for Health Research Evaluation, Trials and Studies Coordinating Centre (NETSCC), University of Southampton<br>Pek, Wei; University of Southampton, Faculty of Medicine<br>Kirkpatrick, Emma ; University of Southampton, Southampton Clinical Trials Unit<br>Ashton-Key, Martin; University of Southampton, NETSCC

**Primary Subject Heading**:	Research methods
Secondary Subject Heading:	Evidence based practice
Keywords:	Randomised Controlled Trials, Pilot studies, Feasibility studies, Health Technology Assessment

SCHOLARONE™ Manuscripts

For peer review only

Title Page

The role of feasibility and pilot studies in randomised controlled trials: a cross-sectional study

Blatch-Jones, Amanda Jane[a*]
Senior Research Fellow

Pek, Wei[b]
Medical student, BM5 Medicine

Kirkpatrick, Emma[c]
Trial Manager

Ashton-Key, Martin[a]
Scientific Director and Consultant in Public Health Medicine

[a]National Institute for Health Research Evaluation, Trials and Studies Coordinating Centre (NETSCC), University of Southampton
Alpha House,
Enterprise Road
Southampton, UK SO16 7NS

[b] Faculty of Medicine
University of Southampton
Southampton General Hospital
Southampton
UK SO16 6YD

[c] Southampton Clinical Trials Unit
University of Southampton
Southampton General Hospital
Southampton
UK SO16 6YD

*Corresponding author's email address: ajy5@soton.ac.uk

For peer review only - http://bmjopen.bmj.com/site/about/guidelines.xhtml

Abstract

Objectives: To assess the value of pilot and feasibility studies to randomised controlled trials (RCTs) funded by the National Institute for Health Research (NIHR) Health Technology Assessment (HTA) programme. To explore the methodological components of pilot / feasibility studies and how they inform full RCTs.

Study design: Cross-sectional study.

Setting: Both groups included NIHR HTA Programme funded studies in the period 1 January 2010-31 December 2014 (decision date). Group 1: Standalone pilot/feasibility studies published in the HTA Journal or accepted for publication. Group 2: all funded RCT applications funded by the HTA Programme, including reference to an internal and/or external pilot/feasibility study. The methodological components were assessed using an adapted framework from a previous study.

Main outcome measures: The proportion of standalone pilot and feasibility studies which recommended proceeding to full trial and what study elements were assessed. The proportion of 'HTA funded' trials which used internal and external pilot and feasibility studies to inform the design of the trial.

Results: Group 1 identified 15 standalone pilot/feasibility studies. Study elements most commonly assessed were *testing recruitment* (100% in both groups), *feasibility* (83%, 100%) and *suggestions for further study/investigation* (83%, 100%). Group 2 identified 161 'HTA funded' applications: 59 cited an external pilot/feasibility study where *testing recruitment* (50%, 73%) and *feasibility* (42%, 73%) were the most commonly reported study elements: 92 reported an internal pilot/feasibility study where *testing recruitment* (93%, 100%) and *feasibility* (44%, 92%) were the most common study elements reported.

Conclusions: 'HTA funded' research which includes pilot and feasibility studies assesses a variety of study elements. Pilot and feasibility studies serve an important role when determining the most appropriate trial design. However, how they are reported and in what context, requires caution when interpreting the findings and delivering a definitive trial.

Keywords: Randomised Controlled Trials, Pilot studies, Feasibility studies, Health Technology Assessment

For peer review only

For peer review only - http://bmjopen.bmj.com/site/about/guidelines.xhtml

Article summary

Strengths and limitations of this study

- This paper assesses the role of pilot and feasibility studies funded by the NIHR HTA programme
- The study found that pilot and feasibility studies share common elements when contributing to the design of a trial
- The study contributes to the growing literature in this area and demonstrates the value of pilot and feasibility studies to the progression to full RCTs
- Although the data covers a five year period, the number of eligible studies are small and only report from one NIHR Programme

Acknowledgements: We would like to acknowledge the NIHR HTA Programme for providing data support during the preliminary stage of the study and the NETSCC Reporting Services team for identifying the relevant applications for the two groups. Professor Jane Blazeby for providing external expert advice during the study.

Ethic statement: This study did not require ethics approval as no patient or clinical data were required.

Funding statement: This study was supported by the NIHR Evaluation, Trials and Studies Coordinating Centre through the Research on Research programme as part of a University of Southampton, Faculty of Medicine, BM5 Medicine, 4th Year project. The views and opinions are those of the authors and do not necessarily reflect those of the Department of Health, or of NETSCC. This research received no specific grant from any funding agency in the public, commercial or not-for-profit sectors.

Contribution of Authors: The study was conceived and designed by MAK, ABJ and EK. The work was undertaken by WP as part of a 4th medical student research project, supervised and supported by MAK, ABJ and EK. Analysis and quality assurance were conducted by WP and MAK and the Access database was prepared by ABJ. All authors read and approved the final manuscript. ABJ is guarantor of the study.

Data sharing: The datasets used and analysed, and anonymised data are available on request from the corresponding author.

Licence for publication: The Corresponding Author has the right to grant on behalf of all authors and does grant on behalf of all authors, a worldwide license to the Publishers and its licensees in perpetuity, in all forms, formats and media (whether known now or created in the future), to i) publish, reproduce, distribute, display and store the Contribution, ii) translate the Contribution into other languages, create adaptations, reprints, include within collections and create summaries, extracts and/or, abstracts of the Contribution, iii) create any other derivative work(s) based on the Contribution, iv) to exploit all subsidiary rights in the Contribution, v) the inclusion of electronic links from the Contribution to third party material where-ever it may be located; and, vi) license any third party to do any or all of the above.

Competing interests: The authors have no competing financial interests; however, ABJ and MAK are employed by the University of Southampton to work for NETSCC. ABJ is employed as the Senior Research Fellow for the Research on Research programme and has worked for NETSCC (and its predecessor organisation) in various roles since 2008. MAK is the scientific

For peer review only - http://bmjopen.bmj.com/site/about/guidelines.xhtml

director at NETSCC and was an editor of the Health Technology Assessment journal. EK is employed by the University of Southampton and works at Southampton's Clinical Trials Unit. EK worked for the Research on Research programme during June 2014 to December 2015. WP was a 4th Year BM5 Medicine student at the University of Southampton.

Abstract word count: 299
Main text word count: 4388

For peer review only

INTRODUCTION

Pilot and feasibility studies have an important role to play in the development of Randomised Controlled Trials (RCTs). If appropriately used, pilot and feasibility studies can provide sufficient methodological evidence about the design planning and justification of a trial. They are often undertaken to inform elements of the main trial design, but they can also be used to reduce or eliminate problems that limit the successful delivery of trials. In 2009, the Lancet published a paper that highlighted the extent to which research is wasted, and that loss is as much as 85% of research investment.[1] Given the cost and time of investment from researchers and major health research funders, there is now a growing demand to assess and examine where improvements need to be made to the design and conduct of trials.[2] Poorly designed trials could include non-reference to a pre-existing systematic literature review or bias generated by inadequate concealment of treatment allocation.[1] Research by Cooper *et al.* has also shown variability between external pilots and the prediction for randomisation and attrition rates.[3] As a result, much attention has primarily focused on the design, conduct and analysis of clinical research to determine where improvements are needed to reduce waste in research.

Over the last ten years, we have seen how pilot and feasibility studies have become an important feature in terms of gathering evidence to inform the development of a full trial. There is now an extension to the Consolidated Standards of Reporting Trials (CONSORT) which provides guidance for pilot and feasibility studies being conducted prior to a main trial.[4] Conducting a pilot or feasibility study to determine any uncertainties prior to the main trial may help to eradicate issues and thus inform the definitive trial. More importantly perhaps, is the role pilot and feasibility studies can have in modifying the design and conduct, and therefore increasing the value of the research, helping to avoid methodological design flaws and reducing the burden of research waste.

Despite the growing importance of pilot and feasibility studies there is still a lack of clarity about the use of the two terms.[5-7] In 2008, the Medical Research Council (MRC) published guidance on developing and evaluating complex interventions to demonstrate the value and importance of pilot and feasibility studies as a key element in the development and evaluation process. However, the guidance did not attempt to explain or provide any definition for the terms 'pilot' and 'feasibility'.[7] It was not until two years later that Thabane *et al.* reviewed the key aspects of pilot studies and provided a detailed account of pilot studies which included a number of definitions.[6] Around the same time (2009), the National Institute for Health Research, Evaluation, Trials and Studies Coordinating Centre (NETSCC) published a support document detailing what feasibility and pilot studies are.

- Feasibility studies are defined as "pieces of research done before a main study in order to answer the question "Can this study be done?". They are used to estimate important parameters that are needed to design the main study…feasibility studies do not evaluate the outcome of interest."
- Pilot studies are defined as "a version of the main study that is run in miniature to test whether the components of the main study can all work together. It is focused on the processes of the main study…it will therefore resemble the main study in many respects."[8]

These definitions have gone some way to aid the understanding of when it is appropriate to do pilot or feasibility studies as part of the definitive trial. These definitions are now widely used across NIHR.

Despite the importance of the role of pilot and feasibility studies in informing RCTs, there is little empirical evidence about the use of these studies in informing future trials. For example, the Lancet series in 2014 did not make reference to the usefulness of pilot and feasibility studies in

For peer review only - http://bmjopen.bmj.com/site/about/guidelines.xhtml

the context of increasing value and reducing waste in research design, conduct or analysis.[9] Lancaster *et al.* and Arain *et al.* provided a methodological framework to assess how pilot studies are used to inform the conduct and reporting of pilot studies.[5 10] Both described the challenges and complexities in the reporting of pilot studies. Arain *et al.* further explored these complexities in relation to feasibility studies and full trials. More recently, research has begun to explore the differences between internal and external pilot studies and their contribution to main trials, and the appropriateness of pilot and feasibility studies for estimating the sample size.[3 11 12]

The aim of this study is to contribute evidence to this important gap in the current literature. The objective of this paper is to describe the process and results of how, and in what way, pilot and feasibility studies have been used to inform full RCTs.

For peer review only

For peer review only - http://bmjopen.bmj.com/site/about/guidelines.xhtml

METHODS

The Health Technology Assessment (HTA) Programme has a long history of commissioning pilot and feasibility studies. Therefore, the published reports (NIHR HTA Journal) of standalone pilot and feasibility studies were examined to determine which elements of research design are most often assessed. Applications for funded HTA trials were also assessed to establish how full trials were informed by previously completed pilot / feasibility studies as well as pilot studies embedded within the trial.

Data source

An assessment of the NIHR HTA Programme over a five-year period (2010-2014) was conducted using two retrospective groups. There were two groups due to the data being homogenous (data for group 1 was taken from the published HTA journal article and data for group 2 was taken from the HTA application form).

In order to identify the included studies for both groups we
1. Reviewed the project title in the application form and the journal article title
2. Reviewed the abstract / executive summary
3. Reviewed the full Journal article or HTA application form

Sample selection

Group 1: Standalone pilot and feasibility studies

Standalone pilot / feasibility studies funded by the HTA Programme with a fund decision date from 01 January 2010 to 31 December 2014, which have published in the HTA Journal or are currently being prepared for publication and have been signed off by the editors (only those in production) were included. The published Journal/approved final version of the published report was used as the source for data extraction. The standalone studies were categorised into 'pilot study', 'feasibility study' or 'both'.

Group 2: Randomised Controlled Trials

Trials funded through the HTA Programme with a fund decision date between 01 January 2010 to 31 December 2014 were included. The application form of a funded trial was used as the source for data extraction. The trials were categorised based on the type of pilot and/or feasibility: 'external / previous pilot study', 'external / previous feasibility study', 'internal pilot study', 'internal feasibility study' or 'other (mixed study)'.

NIHR Evaluation, Trials and Studies Management Information System (NETS MIS) was used to identify the two groups and extract the relevant documents needed for data extraction. Search terms were used to search for relevant data to ensure the feasibility of future replications. The key search terms used were: pilot, feasibility, preliminary work, earlier/previous study.

In addition to the search using key terms, a targeted search was carried out on specific areas of the application form. Focusing on specific areas of the application was relevant in identifying where the elements of the study design would most likely be described in relation to the pilot and/or feasibility study.

Piloting

Data extraction tables for group 1 and group 2 were piloted with an initial sample of 10 studies. No changes were required to the classification system previously adopted by Arain *et al.* as a result of the pilot work.

For peer review only - http://bmjopen.bmj.com/site/about/guidelines.xhtml

Classification systems

The definition of pilot and feasibility studies agreed by NIHR for four Programmes was used for the purpose of this study[8]. These definitions were also used by Arain *et al.*[10]

The classification systems developed by Arain *et al.* and Bugge *et al.* were adapted to determine what elements of a study design were assessed or used to inform the full trial (see *Table 1*).[10 13] In both groups, the elements of the study design were examined in terms of

a. Did the study explicitly state it assessed any of these elements? (yes/no)
b. Were there any recommended changes as a result of the assessment? A yes response was defined as: the authors reported a change / recommendation to be considered but did not necessarily report what that change was. If the authors did not explicitly state a recommendation, it was assumed that no changes were required.

The text pertaining to the pilot and/or feasibility study was also extracted for quality assurance purposes.

Insert Table 1 here

Two additional study elements were included in group two which were not reported in group one. These were '*delivery of intervention*' and '*testing/developing materials*'.

Patient and Public Involvement

There was no patient or public involvement in the design of the study due to the nature of the project (part of a University of Southampton, Faculty of Medicine, BM5 Medicine, 4th Year project). There was no participant recruitment involved in the project, as all data were taken from the published article or the HTA application.

Data quality and assurance

Our approach to quality assurance was guided by the Strengthening the Reporting of Observational Studies in Epidemiology (STROBE), which although designed for observational studies, could be applied to the processes we used in this current study.

For both groups, WP extracted all data and a second person assessed and reviewed the data to ensure the accuracy of data extraction. All of group 1 was assessed followed by 15% of group 2 (purposive sampling of 5% of the group followed by 10% randomly selected application forms). The remaining 85% was subsequently reviewed by MAK to determine the reliability and validity of the data extraction and usability of the adapted template. All disagreements were discussed by the team and were resolved by consensus. Data management was undertaken by WP with support from ABJ.

Data analysis

Data for each study, based on the framework developed by Arain *et al.* (see Table 1), was captured using Microsoft Access 2010 (Microsoft Corporation, Redmond, WA, USA).[10] The study design elements were entered onto an Access form and where a study element was reported a 'yes' response was captured. A separate Access form was developed for each included study for both groups. Both groups were exported into Microsoft Excel 2010 (Microsoft Corporation, Redmond, WA, USA) and then subsequently into Statistical Product and Service Solutions (SPSS) version 22 (IMB Corporation, Armonk, NY, USA). Excel was used to calculate the median and the range for group 1 only. Data were analysed and interpreted using descriptive statistics to determine the frequency of the study design elements and how often changes were recommended for full trials.

For peer review only - http://bmjopen.bmj.com/site/about/guidelines.xhtml

Results

In group 1 we identified 47 published standalone pilot and/or feasibility studies and in group 2 we identified 303 'HTA funded' RCTs during the five-year period (01 January 2010 – 31 December 2014). Fifteen standalone studies were identified as eligible for group 1 and 161 funded HTA applications were identified and eligible for group 2.

Group 1

A total of 47 standalone studies were identified. Thirty-two were excluded on further examination due to not being a pilot or feasibility study (we did not categorise the excluded studies by study design). The remaining 15 studies were categorised into three separate groups (see *Figure 1*). We found that 13 of the 15 study elements included in the adapted framework were assessed in standalone pilot studies compared to nine study elements in feasibility studies.

In this group, it was found that seven studies used the terms "pilot" and "feasibility" interchangeably and it was difficult to determine, even with the NIHR definition, what type of study was undertaken. Therefore, it was not possible to accurately determine which study elements belonged to which, and in some cases the authors described the conduct of both pilot and feasibility work. The team agreed to combine pilot and feasibility together in this instance, which was also found in Arain *et al*.

Insert Figure 1 here

The median number of participants for the standalone studies (n=15) was 46. Of the 15 eligible standalone pilot and/or feasibility studies, the most commonly reported study design element was *testing recruitment*. In all three groups (pilot studies, feasibility studies and pilot/feasibility studies), all 15 studies assessed recruitment (6/6, 2/2 and 7/7 respectively) (see *Table 2*). Half of these also reported recommended changes to recruitment (3/6, 1/2 and 3/7 respectively). Interestingly, both feasibility studies only (2/2) and pilot/feasibility groups (7/7) assessed the need for *further study* and suggested recommended changes (*further study* referred to whether further investigation was required using a large RCT and where future trial data could be of benefit).

Insert Table 2 here

Group 2

A total of 303 'HTA funded' applications were identified. Eighty-two were excluded upon examination as they were not RCTs (for example cohort studies, diagnostic accuracy test studies and we did not categorise the excluded studies by study design) and a further 60 applications were excluded due to not being informed by any external or internal pilot and/or feasibility study. The remaining 161 applications were reviewed and subsequently grouped into five categories (see *Figure 2*).

1. External pilot studies (n=48)
2. External feasibility studies (n=11)
3. Internal pilot studies (n=80)
4. Internal feasibility studies (n=12)
5. Other (n=10)

As the HTA application was used as the source of data extraction, the outcome of the internal pilot/feasibility study was not available (n=92). For the 59 applications where an external pilot / feasibility study was referenced, we found that not all of these studies provided information relating to the number of participants that took part in the pilot/feasibility study. We did not go

For peer review only - http://bmjopen.bmj.com/site/about/guidelines.xhtml

back to the original journal article to retrieve this information. Therefore, it was not appropriate to estimate the median or inter quartile range for this group.
The *others* group comprised applications that were informed by a combination of more than one preliminary study (e.g. internal and/or external pilot study and/or feasibility study). Of those 10 applications,

- Six of the ten were informed by external pilot studies,
- Seven of the ten were informed by external feasibility studies,
- Seven of the ten were informed by internal pilot studies and
- One of the ten was informed by an internal feasibility study.

No further analysis was conducted on these 10 applications due to the diverse nature of the study types in this subgroup.

Insert Figure 2 here

External pilot and feasibility studies

Of the 161 applications, 29.8% (48/161) reported or cited a previous external pilot study not recently done by the applicant and 6.8% (11/161) reported an external feasibility study. For this subset, all of the study elements (n=17) were assessed by external pilot studies but no single study assessed all 17 elements. By comparison, 13 of the 17 study elements were assessed by external feasibility studies (see *Table 3*).

In terms of the study elements, *testing recruitment*, *determining the sample size and numbers available*, and the *feasibility* were the most commonly reported in both external pilot and feasibility studies. The number of reported recommended changes based on the results of the external pilot or feasibility study were however minimal. Although, in some applications it was possible to detect a change, the authors did not explicitly state a recommended change. Therefore, it was not possible to determine whether this was based on the pilot or feasibility study, or some other factor.

Insert Table 3 here

Internal pilot and feasibility studies

Of the 161 applications, 49.7% (80/161) reported an internal pilot study and 7.5% (12/161) reported an internal feasibility study. Due to the source of data extraction (the application form) it was not possible to determine whether the funded internal pilot or feasibility study had made any recommended changes, as the internal study had not yet been conducted.

For the internal studies, we found 14 of the 17 study elements were being assessed by internal pilot studies compared to 10 study elements in feasibility studies. Based on assessment only, the most common study element to be reported was *testing recruitment* (74/80 and 12/12 respectively) and *feasibility* (35/80 and 11/12 respectively) for both internal pilot and feasibility study (see *Table 4*). There were several similarities between a number of study elements assessed by both pilot and feasibility studies.

Insert Table 4 here

For peer review only - http://bmjopen.bmj.com/site/about/guidelines.xhtml

For peer review only

For peer review only - http://bmjopen.bmj.com/site/about/guidelines.xhtml

Discussion

This study found that pilot and feasibility studies do play a role in the development and design of definitive RCTs. In both groups, it was clear that two study elements were commonly assessed: t*esting recruitment* and *feasibility*. This has important implications for the success of a trial, given that many trials struggle with recruitment and often request extensions or become at risk of closure.[14 15] Our findings showed how trials use pilot and/or feasibility studies in an attempt to assess and evaluate prior to a full trial, whether it is likely to be able to recruit its target sample size and whether the study is indeed feasible as a full trial. In both groups, we found pilot studies assessed more study elements than feasibility studies. This also applied to the internal and external studies in group 2; external and internal pilot studies were used to assess more study elements than feasibility studies.

Strengths and weaknesses of the study and in relation to other studies

The main strength of the study was the inclusion of all 'HTA funded' studies over a five-year period. Although the standalone group only included 15 studies, this was as expected. For this group, we identified an increase in almost all of the study elements being assessed compared to earlier work by Arain *et al.*[10] In group two, over half of the 'HTA funded' applications included a pilot and/or feasibility study (internal and/or external) (161/303). Compared to Arain *et al.*[10] the findings were similar for the external pilot and feasibility studies cited in terms of the number of study elements assessed and the number of studies included. For example, *testing recruitment* was the most frequently reported element for pilot studies in both the current study and Arain *et al.*, and d*etermining the sample size and the numbers available* was identical in both studies. However, *randomisation*, *clinical outcomes* and *feasibility* were reported more frequently by Arain *et al.* than the current study. For the external feasibility studies, the current study found more study elements being assessed than that of Arian *et al.* in terms of *testing recruitment*, *determining the sample size and the numbers available*, *randomisation*, *acceptability*, *feasibility* and *follow up/drop out*.

For the internal pilot studies, similar findings were found when comparing Arain *et al.* to the current study: d*etermining the sample size and the numbers available, randomisation* and *clinical outcomes* were assessed more in Arain *et al.* than the current study. As with the internal feasibility studies, we found the current study to report more study elements being assessed than that of Arain *et al.*: *testing recruitment*, *determining the sample size and the numbers available*, *follow up/drop out*, *randomisation*, *acceptability* and *feasibility*. These differences, particularly found with the feasibility studies could be associated with changes over time in the use and understanding of feasibility studies.

This study relied on an adapted version of the Arain *et al.* framework. As some of the study elements were expanded and new ones were added a direct comparison with Arain *et al.* findings is limited.[10] Given the subjective nature of some of the study elements, we chose to quality assure all data to eradicate and reduce any known errors. Since the analysis was based explicitly on the reporting of the applicants, and did not include any subjective account or interpretation of what was reported, we may have under reported the number of study elements assessed and/or recommended.

We also noted a mismatch in numbers between those assessing study elements and those where recommendations were made in group one. This was due, in part, to how each study element was reported by the applicants. For example if a study did not specify that they had assessed these elements but made recommendation for changes, we only inferred that they assessed it, but it could not be recorded in the data, hence the mismatch in the findings. This

For peer review only - http://bmjopen.bmj.com/site/about/guidelines.xhtml

does however highlight the importance of clearly reporting how, what and where the pilot and/or feasibility study had an impact on the design of the definitive trial.

Implications

The level of appropriateness in the reporting of pilot and feasibility studies could largely be affected by the lack of clarity and awareness of the different study requirements. Despite the growing literature on improving the quality of research to reduce waste in research, there is limited literature pertaining to how pilot and feasibility studies fit into this agenda for change. From what literature there is on pilot and feasibility studies, there is still some confusion about when, why and how it is appropriate to conduct a pilot and/or feasibility study. The findings in this study, even with the use of a well-defined definition by NIHR, still found evidence where applicants did not adhere to the HTA definitions for "pilot" and "feasibility" study on research applications. The terminology is still being used interchangeably. Although the commentary on pilot studies by Thabane *et al.* gives a detailed account of the appropriateness of why and how to conduct a pilot study, a comparison with feasibility studies is lacking.[6] It would be helpful to have a more formal distinction between these two terminologies as suggested by Arain *et al.* A recent study by Eldridge *et al.* goes some way to rectify this by developing a conceptual framework for defining pilot and feasibility studies.[16] The conceptual framework shows promising results, by being compatible with the MRC guidance on complex interventions,[7] and their descriptor of pilot studies is similar to that of the NIHR definition. However, it is important to note that the Eldridge *et al.* conceptual framework is slightly different from that adopted by the NIHR.[16] The clear lack of dichotomy between pilot and feasibility studies is an area for future consideration, not only for funders to encourage more conformity to the published definitions, but for researchers to make better use of the existing literature to better understand the distinction between pilot and feasibility studies.

Having clear definitions of when to use pilot and feasibility studies is important both in terms of their purpose and for clarifying progression to a full trial. However, it is also important to note the limitations of pilot and feasibility studies and when it is not appropriate to conduct this type of study. Pilot and feasibility studies provide valuable information to inform the design of any subsequent definitive study including for example, approaches to consent, willingness to recruit and randomisation, and adherence to any proposed intervention. Although they are not usually sufficiently powered to provide estimates of effect size they can provide data that may be useful in helping define the final size of any subsequent study. However, how they are reported, and in what context, requires caution especially when interpreting the findings and extrapolating these to the delivery of a definitive trial.[11 17 3 12]

Conclusion and recommendations

'HTA funded' research which is inclusive of pilot and feasibility studies is very likely to assess a variety of study elements, which have been evidence-based through this current study using an adapted version of Arain *et al.* framework.[10] However, not reviewing the impact of the preliminary work once the trial commences, we have no way of knowing whether the pilot and/or feasibility studies recommendations were instrumental in the successful completion of the trial. If we are able to demonstrate the value of pilot and feasibility studies we need to place greater emphasis on not only their role in the design stage of a trial but also how this preliminary work

For peer review only - http://bmjopen.bmj.com/site/about/guidelines.xhtml

contributed favourably, or not, to the completion of the definitive trial. The internal pilot and/or feasibility studies reported in group 2 could be used for the basis of continued work in this area. By following up on this group we would be able to analyse the successful delivery of the definitive trial and whether the preliminary work had any bearing on this success.

Recommendations include a larger sample of studies across other UK health research funding agencies to determine the frequency and importance of those study elements reported here. A further assessment between the study elements noted in the pilot and feasibility studies and how this impacted on the eventual design and conduct of the definitive trial would certainly add value. This could be achieved by prospectively evaluating the ongoing use of pilot and feasibility studies in group two (specifically the internal pilot and/or feasibility studies) as well as future funded applications to the HTA programme. Highlighting the need for better reporting of pilot and feasibility studies should be regarded as relevant to all research funding bodies. And as such, better guidelines for the design, conduct, analysis and reporting of pilot and feasibility studies are still needed.

Future work could therefore include widening the study outcomes presented here to other NIHR funded research programmes. Funders might want to consider the use of Arain *et al.* framework when considering the funding of pilot / feasibility studies. Where appropriate this could contribute to maximising the benefit of research and reducing the extent to which research is wasted. If we find ways to appropriately address the flaws detected at the design and conduct stages of research, then we could start to see how research adds value and reduces the amount of research waste. In order to achieve this, we need clearly defined terminology which is inclusive of funding agencies and researchers' perspective; empirical evidence on the reporting and appropriate use of pilot and feasibility studies, in terms of favourable study elements and; an evaluation of the contribution to definitive trial outcomes.

For peer review only - http://bmjopen.bmj.com/site/about/guidelines.xhtml

Table 1: Elements of a study design adapted from Arain *et al.*

**The methodological components included as reported by and included from Arain et al:**
**Methods related**
Testing Recruitment
Determining the sample size / numbers available
Follow up / dropout
Hypothesis testing
Resources
Randomisation
Blinding
Outcome measures
Control group
Data collection
Further study suggested
**Intervention related**
Dose / efficacy / safety
Clinical outcomes
Acceptability
Feasibility
In addition to the above, group 2 included:
Delivery of the intervention
Testing/developing materials

For peer review only

For peer review only - http://bmjopen.bmj.com/site/about/guidelines.xhtml

Table 2: Group 1 - Study elements captured in pilot studies, feasibility studies and pilot/feasibility studies

	Pilot studies only (n=6)			Feasibility studies only (n=2)			Pilot/Feasibility studies (n=7)		
**Study elements**	**Assessed (A): Number (%)**	**Recommended changes (RC): Number (%)**	**A and RC: Number (%)**	**Assessed (A): Number (%)**	**Recommended changes (RC): Number (%)**	**A and RC: Number (%)**	**Assessed (A): Number (%)**	**Recommended changes (RC): Number (%)**	**A and RC: Number (%)**
Testing recruitment	6 (100.0)	3 (50.0)	3 (50.0)	2 (100.0)	1 (50.0)	1 (50.0)	7 (100.0)	3 (42.9)	3 (42.9)
Determining Sample Size and/or number available	5 (83.3)	1 (16.6)	0	1 (50.0)	2 (100.0)	1 (50.0)	5 (71.4)	1 (14.3)	1 (14.3)
Follow up/dropout	4 (66.6)	3 (50.0)	3 (50.0)	0	1 (50.0)	0	5 (71.4)	2 (28.6)	1 (14.3)
Hypothesis testing	2 (33.3)	0	0	1 (50.0)	0	0	1 (14.3)	0	0
Resources	4 (66.6)	3 (50.0)	2 (33.3)	2 (100.0)	0	0	5 (71.4)	1 (14.3)	1 (14.3)
Randomisation	4 (66.6)	0	0	0	0	0	6 (85.7)	1 (14.3)	1(14.3)
Blinding	0	0	0	0	0	0	2 (28.6)	0	0
Outcome measures	5 (83.3)	4 (66.6)	4 (66.6)	2 (100.0)	1 (50.0)	1 (50.0)	6 (85.7)	4 (57.1)	3 (42.9)
Control group	0	0	0	0	0	0	1 (14.3)	0	0
Data collection	3 (50.0)	0	0	0	1 (50.0)	0	3 (42.9)	1 (14.3)	0
Clinical outcomes	3 (50.0)	2 (33.3)	1 (16.6)	1 (50.0)	0	0	3 (42.9)	0	0
Dose/efficacy/safety	2 (33.3)	1 (16.6)	1 (16.6)	0	0	0	2 (28.6)	0	0
Acceptability	4 (66.6)	0	0	1 (50.0)	0	0	6 (85.7)	0	0
Feasibility	5 (83.3)	0	0	2 (100.0)	0	0	7 (100.0)	0	0
Suggests further study	5 (83.3)	4 (66.6)	4 (66.6)	2 (100.0)	2 (100.0)	2 (100.0)	7 (100.0)	7 (100.0)	7 (100.0)
Median number of participants (IQR) [Range]	47.5 (39.25-85) [21-99]			14 (7-21) [0-28]			58 (35.5-173) [29-313]		

For peer review only - http://bmjopen.bmj.com/site/about/guidelines.xhtml

Table 3: Group 2 - Study elements captured in external pilot and feasibility studies

Study elements	External pilot study (n=48)		External feasibility study (n=11)	
	Assessed Number (%)	Recommended changes Number (%)	Assessed Number (%)	Recommended changes (n)
Testing recruitment	24 (50.0)	3 (6.3)	8 (72.7)	0
Determining sample size and/or number available	24 (50.0)	1 (2.1)	4 (36.4)	1 (9.1)
Follow up/dropout	16 (33.3)	0	3 (27.3)	0
Hypothesis testing	10 (20.8)	0	2 (18.2)	0
Resources	2 (4.2)	0	1 (9)	0
Randomisation	7 (14.6)	0	3 (27.3)	0
Blinding	4 (8.3)	1 (2.1)	0	0
Outcome measures	10 (20.8)	1 (2.1)	1 (9.1)	0
Control group	3 (6.3)	0	0	0
Data collection	6 (12.5)	0	2 (18.2)	0
Clinical outcomes	12 (25.0)	0	1 (9.1)	0
Dose/efficacy/safety	14(29.2)	1 (2.1)	0	0
Acceptability	17 (35.4)	0	4 (36.4)	0
Feasibility	20 (41.7)	0	8 (72.7)	0
Suggests further study	8 (16.6)	1 (2.1)	1 (9.1)	0
Delivery of intervention	8 (16.6)	2 (4.2)	0	0
Testing/developing materials	3 (6.3)	0	1 (9.1)	0

Table 4: Group 2 - Study elements captured in internal pilot and feasibility studies

Study elements	Internal pilot study (n=80) Assessed No. (%)	Internal feasibility study (n=12) Assessed No. (%)
Testing recruitment	74 (92.5)	12 (100.0)
Determining sample size and/or number available	21 (26.3)	4 (33.3)
Follow up/dropout	28 (35.0)	5 (41.7)
Hypothesis testing	0	0
Resources	3 (3.8)	1 (8.3)
Randomisation	27 (33.8)	4 (33.3)
Blinding	2 (2.5)	0
Outcome measures	16 (20.0)	2 (16.7)
Control group	0	0
Data collection	21 (26.3)	2 (16.7)
Clinical outcomes	1 (1.3)	0
Dose/efficacy/safety	5 (6.3)	1 (8.3)
Acceptability	21 (26.3)	7 (58.3)
Feasibility	35 (43.8)	11 (91.7)
Suggests further study	0	0
Delivery of intervention	7 (8.8)	0
Testing/developing materials	7 (8.8)	0

For peer review only - http://bmjopen.bmj.com/site/about/guidelines.xhtml

References

1. Chalmers I, Glasziou P. Avoidable waste in the production and reporting of research evidence. *The Lancet* 2009;374(9683):86-89. doi: doi: DOI: 10.1016/S0140-6736(09)60329-9
2. Moher D, Glasziou P, Chalmers I, et al. Increasing value and reducing waste in biomedical research: who's listening? *The Lancet* 2016;387(10027):1573-86. doi: https://doi.org/10.1016/S0140-6736(15)00307-4
3. Cooper CL, Whitehead A, Pottrill E, et al. Are pilot trials useful for predicting randomisation and attrition rates in definitive studies: A review of publicly funded trials. *Clinical Trials* 2018:1740774517752113.
4. Eldridge SM, Chan CL, Campbell MJ, et al. CONSORT 2010 statement: extension to randomised pilot and feasibility trials. *BMJ* 2016;2(1):64.
5. Lancaster GA, Dodd S, Williamson PR. Design and analysis of pilot studies: recommendations for good practice. *Journal of Evaluation in Clinical Practice* 2004;10(2):307-12. doi: 10.1111/j..2002.384.doc.x
6. Thabane L, Ma J, Chu R, et al. A tutorial on pilot studies: the what, why and how. *BMC medical research methodology* 2010;10(1):1.
7. Craig P, Dieppe P, Macintyre S, et al. Developing and evaluating complex interventions: The new Medical Research Council guidance. *International Journal of Nursing Studies* 2013;50(5):587-92. doi: 10.1016/j.ijnurstu.2012.09.010
8. NIHR. Feasibility and Pilot studies https://www.nihr.ac.uk/funding-and-support/documents/funding-for-research-studies/research-programmes/PGfAR/Feasibility%20and%20Pilot%20studies.pdf 2018 [accessed 6 Feb 2018.
9. Ioannidis JP, Greenland S, Hlatky MA, et al. Increasing value and reducing waste in research design, conduct, and analysis. *Lancet* 2014;383(9912):166-75. doi: 10.1016/S0140-6736(13)62227-8
10. Arain M, Campbell MJ, Cooper CL, et al. What is a pilot or feasibility study? A review of current practice and editorial policy. *BMC medical research methodology* 2010;10:67. doi: 10.1186/1471-2288-10-67
11. Whitehead AL, Julious SA, Cooper CL, et al. Estimating the sample size for a pilot randomised trial to minimise the overall trial sample size for the external pilot and main trial for a continuous outcome variable. *Statistical methods in medical research* 2016;25(3):1057-73.
12. Billingham SA, Whitehead AL, Julious SA. An audit of sample sizes for pilot and feasibility trials being undertaken in the United Kingdom registered in the United Kingdom Clinical Research Network database. *BMC medical research methodology* 2013;13(1):104.
13. Bugge C, Williams B, Hagen S, et al. A process for Decision-making after Pilot and feasibility Trials (ADePT): development following a feasibility study of a complex intervention for pelvic organ prolapse. *Trials* 2013;14:353. doi: 10.1186/1745-6215-14-353
14. Raftery J, Young A, Stanton L, et al. Clinical trial metadata: defining and extracting metadata on the design, conduct, results and costs of 125 randomised clinical trials funded by the National Institute for Health Research Health Technology Assessment programme. *Health Technology Assessment* 2015;19(11):1-166.
15. Bower P, Brueton V, Gamble C, et al. Interventions to improve recruitment and retention in clinical trials: a survey and workshop to assess current practice and future priorities. *Trials* 2014;15(1):399.
16. Eldridge SM, Lancaster GA, Campbell MJ, et al. Defining feasibility and pilot studies in preparation for randomised controlled trials: development of a conceptual framework. *PLoS One* 2016;11(3):e0150205.

For peer review only - http://bmjopen.bmj.com/site/about/guidelines.xhtml

17. Vogel S, Draper-Rodi J. The importance of pilot studies, how to write them and what they mean. *International Journal of Osteopathic Medicine* 2017;23:2-3. doi: 10.1016/j.ijosm.2017.02.001

For peer review only

For peer review only - http://bmjopen.bmj.com/site/about/guidelines.xhtml

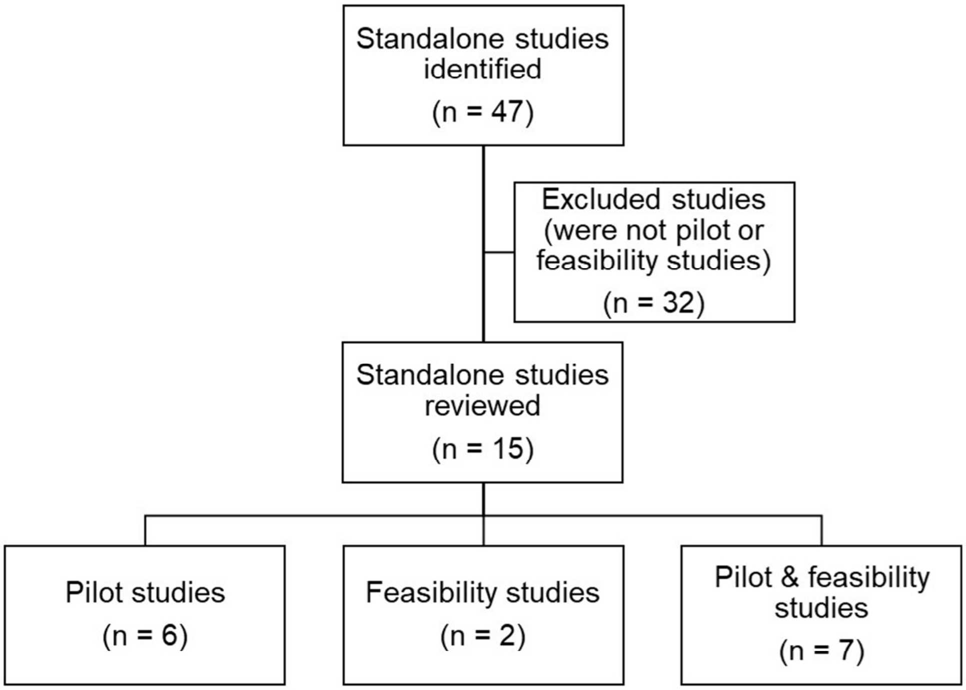

Figure 1: The number of studies identified, excluded and categorised for Cohort 1

Figure 1: The number of studies identified, excluded and categorised for Cohort 1

165x126mm (300 x 300 DPI)

For peer review only - http://bmjopen.bmj.com/site/about/guidelines.xhtml

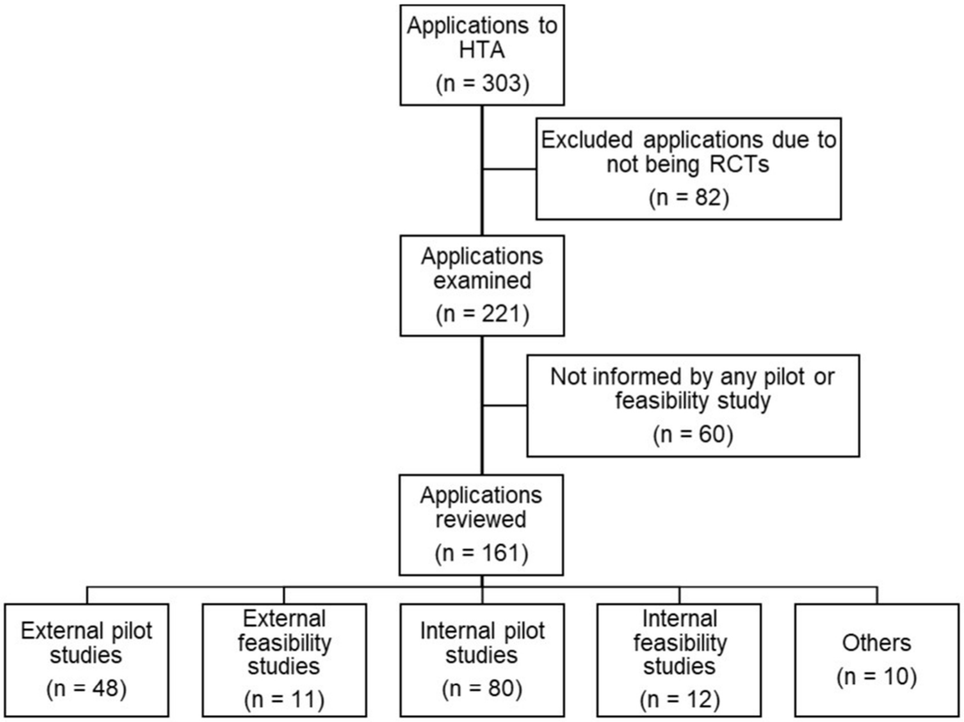

Figure 2: Flow chart showing the number of HTA funded applications for Cohort 2

Figure 2: Flow chart showing the number of HTA funded applications for Cohort 2

147x116mm (300 x 300 DPI)

STROBE Statement—Checklist of items that should be included in reports of ***cohort studies***

	Item No	Recommendation	Page No.
**Title and abstract**	1	(*a*) Indicate the study's design with a commonly used term in the title or the abstract	1
		(*b*) Provide in the abstract an informative and balanced summary of what was done and what was found	2
**Introduction**			
Background/rationale	2	Explain the scientific background and rationale for the investigation being reported	5
Objectives	3	State specific objectives, including any prespecified hypotheses	6
**Methods**			
Study design	4	Present key elements of study design early in the paper	7
Setting	5	Describe the setting, locations, and relevant dates, including periods of recruitment, exposure, follow-up, and data collection	7
Participants	6	(*a*) Give the eligibility criteria, and the sources and methods of selection of participants. Describe methods of follow-up	7
		(*b*) For matched studies, give matching criteria and number of exposed and unexposed	NA
Variables	7	Clearly define all outcomes, exposures, predictors, potential confounders, and effect modifiers. Give diagnostic criteria, if applicable	NA
Data sources/ measurement	8*	For each variable of interest, give sources of data and details of methods of assessment (measurement). Describe comparability of assessment methods if there is more than one group	7,8
Bias	9	Describe any efforts to address potential sources of bias	NA
Study size	10	Explain how the study size was arrived at	7
Quantitative variables	11	Explain how quantitative variables were handled in the analyses. If applicable, describe which groupings were chosen and why	NA
Statistical methods	12	(*a*) Describe all statistical methods, including those used to control for confounding	NA
		(*b*) Describe any methods used to examine subgroups and interactions	NA
		(*c*) Explain how missing data were addressed	NA
		(*d*) If applicable, explain how loss to follow-up was addressed	NA
		(*e*) Describe any sensitivity analyses	NA
**Results**			
Participants	13*	(a) Report numbers of individuals at each stage of study—eg numbers potentially eligible, examined for eligibility, confirmed eligible, included in the study, completing follow-up, and analysed	9,10, Fig1, Fig2
		(b) Give reasons for non-participation at each stage	9,10, Fig1, Fig2
		(c) Consider use of a flow diagram	Fig1, Fig2
Descriptive data	14*	(a) Give characteristics of study participants (eg demographic, clinical, social) and information on exposures and potential confounders	9,10
		(b) Indicate number of participants with missing data for each variable of interest	NA
		(c) Summarise follow-up time (eg, average and total amount)	NA
Outcome data	15*	Report numbers of outcome events or summary measures over time	NA

For peer review only

Main results	16	(*a*) Give unadjusted estimates and, if applicable, confounder-adjusted estimates and their precision (eg, 95% confidence interval). Make clear which confounders were adjusted for and why they were included	NA
		(*b*) Report category boundaries when continuous variables were categorized	NA
		(*c*) If relevant, consider translating estimates of relative risk into absolute risk for a meaningful time period	NA
Other analyses	17	Report other analyses done—eg analyses of subgroups and interactions, and sensitivity analyses	NA
**Discussion**			
Key results	18	Summarise key results with reference to study objectives	11
Limitations	19	Discuss limitations of the study, taking into account sources of potential bias or imprecision. Discuss both direction and magnitude of any potential bias	11
Interpretation	20	Give a cautious overall interpretation of results considering objectives, limitations, multiplicity of analyses, results from similar studies, and other relevant evidence	11
Generalisability	21	Discuss the generalisability (external validity) of the study results	11-13
**Other information**			
Funding	22	Give the source of funding and the role of the funders for the present study and, if applicable, for the original study on which the present article is based	3

*Give information separately for exposed and unexposed groups.

Note: An Explanation and Elaboration article discusses each checklist item and gives methodological background and published examples of transparent reporting. The STROBE checklist is best used in conjunction with this article (freely available on the Web sites of PLoS Medicine at http://www.plosmedicine.org/, Annals of Internal Medicine at http://www.annals.org/, and Epidemiology at http://www.epidem.com/). Information on the STROBE Initiative is available at http://www.strobe-statement.org.

For peer review only

For peer review only - http://bmjopen.bmj.com/site/about/guidelines.xhtml
